# Proteomic Investigation Reveals Eukaryotic Translation Initiation Factor 5A Involvement in Porcine Reproductive and Respiratory Syndrome Virus Infection *in vitro*

**DOI:** 10.3389/fvets.2022.861137

**Published:** 2022-04-13

**Authors:** Huawei Li, Bo Wan, Dawei Jiang, Pengchao Ji, Mengmeng Zhao, Xinfeng Li, Rui Li, Songlin Qiao

**Affiliations:** ^1^Henan Key Laboratory of Innovation and Utilization of Unconventional Feed Resources, Henan University of Animal Husbandry and Economy, Zhengzhou, China; ^2^College of Veterinary Medicine, Henan Agricultural University, Zhengzhou, China; ^3^School of Life Science and Engineering, Foshan University, Foshan, China; ^4^Key Laboratory of Animal Immunology of the Ministry of Agriculture, Henan Provincial Key Laboratory of Animal Immunology, Henan Academy of Agricultural Sciences, Zhengzhou, China

**Keywords:** proteome, eIF5A, PRRSV, infection, PAMs

## Abstract

Porcine reproductive and respiratory syndrome virus (PRRSV), one of the most serious animal pathogens in the world, has caused enormous global swine industry losses. An in-depth investigation of the PRRSV-host interaction would be beneficial for preventing and controlling PRRSV infections and transmission. In this study, we performed label-free quantitative proteomic assays to investigate proteome dynamics of porcine alveolar macrophages (PAMs) during infection with highly pathogenic PRRSV (HP-PRRSV) strain HN07-1. Analysis of the results led to identification of 269 significantly differentially expressed host cellular proteins, of which levels of proteins belonging to the eukaryotic translation initiation factor (eIF) family were found to be decreased in abundance in HP-PRRSV-infected PAMs. Furthermore, knockdown of eIF5A expression was demonstrated to markedly suppress HP-PRRSV propagation, as reflected by reduced progeny virus titers *in vitro*. These results highlight the importance of eIF5A in PRRSV infection, while also demonstrating that PAMs down-regulate eIF5A expression as a host cell antiviral strategy. Results of the current study deepen our understanding of PRRSV pathogenesis and provide novel insights to guide development of effective strategies to combat the virus.

## Introduction

Porcine reproductive and respiratory syndrome (PRRS), a highly contagious disease caused by PRRS virus (PRRSV) infection, leads to reproductive disorders in sows and respiratory symptoms in pigs of all ages (1). PRRS was first discovered in North America in 1987 before it spread around the world, to cause substantial economic swine industry losses (2). In 2006, a severe epidemic of highly pathogenic (HP)-PRRS occurred in China that induced high fever in pigs and was associated with high mortality regardless of age. Importantly, the viral strain that was responsible for the epidemic possessed a 30-amino-acid discontinuous deletion within PRRSV non-structural protein 2 (nsp2) (3, 4).

PRRSV belongs to the family *Arteriviridae* (genus *Betaarterivirus*) within the taxonomic order *Nidovirales* and possesses a single-stranded positive-sense RNA genome of 15.4 kb (5). All PRRSV isolates are classified into two genotypes: PRRSV-1 and PRRSV-2 (6). PRRSV-2 strains were predominant in China. The cell tropism of PRRSV, which is highly limited, includes porcine alveolar macrophages (PAMs) that act as primary host cells to support viral infection (7). Importantly, PRRSV infection is a complex process such that mechanisms associated with PRRSV infection have not yet been fully clarified in spite of intensive research efforts.

Quantitative proteomic techniques, which are classified as label-based and label-free methods, have been used to research viral infections such as influenza virus (8), human respiratory syncytial virus (9), hepatitis B virus (10), porcine circovirus type 2 (11), foot and mouth disease virus (12), infectious bursal disease virus (13), African swine fever virus (14, 15). Consequently, results of these studies have created a foundation of knowledge on which to build future investigations to better understand pathogenesis of other viruses, including PRRSV.

In recent years, researchers have studied PRRSV infection using quantitative proteomic approaches. For example, Fang *et al*. (16) used an acetylation-based antibody enrichment technique and a tandem mass tag label high-affinity purification liquid chromatography-mass spectrometry (LC-MS/MS) method to study acetylome regulation of antiviral activities in PRRSV-infected PAMs. A few years earlier, Zhang et al. (17) had generated a broad-spectrum ubiquitination modification map of PRRSV-infected PAMs using ubiquitination antibody enrichment in combination with MS technology. Slightly earlier, Li et al. (18) conducted label-free quantitative proteomics to detect differentially secreted proteins in supernatants of PRRSV-infected PAMs and compared the results to those obtained for supernatants of uninfected PAMs. These studies not only enhanced our understanding of PRRSV infection, but also identified potential targets of antiviral drugs.

In order to better understand the proteome alterations of host cells during PRRSV infection, a label-free quantitative approach coupled with LC-MS/MS was applied to analyze the altered proteins in HP-PRRSV HN07-1-infected PAMs. The results of these experiments revealed that at 24 h post-infection (hpi), expression levels of 269 host cellular proteins were found to be significantly altered. Subsequent bioinformatic analyses revealed that these differentially expressed proteins were enriched for functional terms corresponding to several biological processes and KEGG pathways. Interestingly, expression levels of translation-related proteins, including eukaryotic translation initiation factors (eIFs), were significantly down-regulated after HP-PRRSV HN07-1 infection, with one such factor, eIF5A, found to be essential for viral replication. The results of this work uncovered for the first time the importance of eIF5A during virus infection, while also providing insights into host-pathogen interactions that occur during PRRSV infection to guide future development of effective antiviral strategies.

## Materials and Methods

### Cells and Virus

PAMs were collected from 4-week-old pathogen-free piglets that had been previously confirmed to be uninfected with PRRSV, porcine circovirus type 2, foot-and-mouth disease virus, pseudorabies virus, classical swine fever virus, and porcine parvovirus. The PAMs collection procedure was approved by the Ethical and Animal Welfare Committee of the Key Laboratory of Animal Immunology of the Ministry of Agriculture of China (permit no. 2018005). Collected PAMs were cultured in RPMI-1640 medium (Hyclone, China) containing 10% fetal bovine serum (FBS, Gibco, USA), 100 μg/mL streptomycin, and 100 units/mL penicillin (Hyclone, China) in a humidified incubator maintained at 37°C with 5% CO_2_. MARC-145 cells were purchased from ATCC and passaged in our laboratory. CRL-2843-CD163 cells were obtained from Professor Enmin Zhou of the College of Veterinary Medicine, Northwest Agriculture and Forestry University. The HP-PRRSV HN07-1 strain (GenBank: KX766378.1) that was used in this study was isolated and identified by the Key Laboratory of Animal Immunology of the Ministry of Agriculture, Henan Provincial Key Laboratory of Animal Immunology, Henan Academy of Agricultural Sciences (19).

### Virus Inoculation

PAMs were infected with HP-PRRSV HN07-1 at a multiplicity of infection (MOI) of 0.1 then the cells were fixed in ice-cold 95% methyl alcohol at 12, 24, 36, and 48 hpi. Next, cells were blocked in 5% skim milk in phosphate buffer solution with Tween-20 (PBST) at 4°C for 12 h. Thereafter, an immunofluorescence assay (IFA) was conducted to detect viral propagation at different time points post-infection using anti-PRRSV nucleocapsid (N) protein antibody as a probe (GeneTex, USA). Thereafter, cells were incubated with fluorescein isothiocyanate (FITC)-labeled goat-anti-mouse IgG (Sigma, USA) as secondary antibody. After incubation with primary and secondary antibodies, the cells were washed with PBST then 4′,6-diamidino-2-phenylindole-dihydrochloride (DAPI, Beyotime, China) was added to cells followed by incubation at room temperature for 30 min to stain the nuclei. Next, an OLYMPUS IX 81 confocal microscope equipped with a digital camera was used to capture the fluorescent images. Then the one-step growth curve of HP-PRRSV HN07-1 in PAMs was plotted based on virus titers obtained at 12, 24, 36, 48, 60, and 72 hpi.

### Sample Preparation, Protein Isolation, and Protein Digestion

PRRSV-infected and uninfected PAMs were gently washed with PBS, then were treated with 0.25% trypsin-EDTA (Solarbio, China) for 2 min. Next, cell suspensions were centrifuged at 1,200 rpm for 6 min then the cells were stored at −80°C until needed for further investigations. Protein extractions from PAMs were conducted as follows: frozen protein pellets were homogenized in lysis buffer (8 M urea, 2 M thiourea, 4% CHAPS, 20 mM Tris base, 30 mM dithiothreitol (DTT), and 2% Bio-Lyte) on ice. After that, samples were subjected to ultrasonic treatment followed by centrifugation at 13,500 rpm at 4°C for 20 min. Thereafter, each supernatant was removed then precipitated with ice-cold acetone at −20°C for 30 min then centrifuged twice (13,500 rpm for 10 min at 4°C) to pellet the proteins. Next, each pellet was collected and dissolved in 40 mM NH_4_HCO_3_ in which DTT was added to a final concentration of 100 mM, and iodoacetamide was also added to a final concentration of 50 mM. Thereafter, reductive alkylation was allowed to proceed for 1 h in dark at room temperature. Protein concentrations were quantified using the Bradford assay (20).

To conduct protein digestions, denatured proteins were reduced in 100 mM DTT and alkylated with 50 mM iodoacetamide to prevent reformation of disulfide bonds. Next, samples were digested with sequencing grade modified trypsin (Promega, USA) then were incubated at 37°C for 14 h. Finally, peptides were pooled and dried using a Speed-Vac system (RVC 2–18, Marin Christ, Germany) then were analyzed via MS/MS.

### LC–MS/MS Analysis

Each dried sample was dissolved in 1 formic acid aqueous solution and centrifuged at 14,000 rpm for 20 min at 4°C. Next, each supernatant was gently transferred to a clean tube to avoid creation of bubbles. Prior to MS analysis, digested peptides were suspended in 15 μL of 0.1% formic acid then 10 μL of each peptide sample was subjected to LC-MS/MS analysis using an Easy nLC1000 System (Thermo Fisher Scientific, USA) coupled to a Q Exactive Orbitrap Spectrometer (Thermo Fisher Scientific) that was equipped with a nanoelectrospray ion source (capillary temperature 275°C, spray voltage 2.3 kV, and S-Lens RF 55%). After addition of loading solvent (2% acetonitrile and 0.1% formic acid in H_2_O) to the tryptic digests, the samples were loaded onto an Easy-Spray column filled with 2 μm C18 resin (75 μm × 50 cm, 100 Å, Thermo Fisher Scientific). Peptides were separated over a period of 130 min using a gradient consisting of 3 to 30% acetonitrile (containing 0.1% formic acid) using an analytical column packed with 3 μm C18 (75 μm × 15 cm, 100 Å, Thermo Fisher Scientific). The mass spectrometer was tuned to positive ion mode, MS scan control was maintained using Xcalibur software 2.2 (Thermo Fisher Scientific), MS data acquisition was data-dependent, repetition count was set to 1, exclusion duration was set to 30 s, and dynamic exclusion was enabled. MS1 precursor scan (m/z 300–2,000) acquisition was carried out in the orbitrap with a nominal resolution of 30,000 at m/z 400. Next, MS/MS fragmentation of the top 20 most intense multiply charged precursor ions was conducted using higher energy collisional dissociation with 35% normalized fragmentation energy. MS2 scans (m/z 100–2,000) were performed using the orbitrap mass analyzer at a resolution setting of 15,000 at m/z 400 and a starting m/z setting of 100.

### Data Analysis and Protein Quantification

MS raw data files were retrieved using Xcalibur 2.2 (Thermo Fisher Scientific) and searched using PEAKS 7.5 against the *Sus Scrofa* database, which contained 38,431 entries when it was downloaded in July 2020 from the NCBI-ref database (21). Search parameters were set as follows: parent mass error tolerance of 20.0 ppm, fragment mass error tolerance of 0.05 Da, enzyme was trypsin. No specific cleavage site was selected for the peptide, maximum missed cleavages per peptide was set to 2, carbamidomethyl (C, +57.02) was selected as the fixed modification, and oxidation (M, +15.99) was selected as the variable modification. Each peptide had at most three types of posttranslational modifications. Protein selection parameters were as follows: false discovery rate (FDR) was ≤1.0% (-10l g *P* ≥ 20.0) and unique peptide with one spectrum was ≥1. The results of database retrieval were quantitatively analyzed using PEAKS Q and the peptide rate was calculated according to peak area. The conditions were set as follows: retention time shift tolerance of 1 min, mass error tolerance of 15 ppm, unique peptide ≥1, charge between 2 and 8, fold change of proteins and peptides was ≥1.5, and significance of ≥3 (*P* ≤ 0.05).

### Bioinformatics Analysis

All identified proteins were used as inputs for functional analysis using ClueGO V2.1.7, a Cytoscape plug-in (http://www.ici.upmc.fr/cluego/), that comprehensively identifies proteins that are involved in various biological signal pathways and protein interactions (22). Protein FASTA files were blasted against the *Sus scrofa* database using GI numbers. Right-sided hypergeometric enrichment was conducted as a statistical test using the Bonferroni step-down correction method (*P* ≤ 0.05) using parameters of gene ontology score range of three to eight, Kappa threshold set to 0.4, and initial group size set to one. Protein-protein interaction (PPI) networks were constructed using another Cytoscape plug-in, GeneMANIA (23), which uses many functional association data, including protein and genetic interactions.

### Real-Time PCR (RT-PCR)

Samples of total RNA of mock- and PRRSV-infected PAMs were prepared using TRIzol reagent (Invitrogen, USA) at 12 and 24 hpi, then cDNAs were generated from total RNA preparations using a reverse transcriptase kit according to the manufacturer's instructions (Takara, Japan). β-Actin served as an internal reference to normalize the data. Primer sequences are listed in Supplementary Table 1. RT-PCR assays were conducted using an Applied Biosystems 7,500 Fast RT-PCR System with 20 μL reactions (performed in triplicate) prepared that contained SYBR Green Premix 10 μL (ROCHE, Switzerland), 0.6 μL of each primer, and 6.8 μL of H_2_O. Each experiment was performed independently three times.

### UV-Inactivation of PRRSV

The virus solution was irradiated by exposure to ultraviolet light of wavelength 254 nm that was emitted by a low-intensity ultraviolet lamp (120 mJ/cm^2^). Irradiation was conducted at room temperature for 30 min to inactivate the virus; the effectiveness of inactivation was assessed using RT-PCR.

### Western Blot (WB)

Mock-infected, PRRSV-infected, and UV-inactivated PRRSV-treated PAMs were harvested at 0, 6, 12, and 24 hpi then the cells were lysed in RIPA buffer (Solarbio) containing 1% PMSF (Solarbio) for 30 min on ice followed by measurement of protein concentrations. For WB analysis, cell lysates containing equivalent concentrations of total protein were subjected to 12% SDS-PAGE then the separated proteins were transferred to 0.45 μm polyvinylidene difluoride (PVDF) membranes (Millipore, USA). After membranes were blocked in 5% skim milk at 4°C overnight, membranes were incubated with polyclonal antibodies specific for myxovirus-resistant protein 1 (Mx1) (Proteintech, China), tetratricopeptide repeats 3 (IFIT3) (Proteintech), PRRSV N (GeneTex, USA), or monoclonal antibody (mAb) specific for signal transducer and activator of transcription 1 (STAT1), eIF5A, eIF4E, eIF4H, 4EBP1, β-Actin (Cell Signaling Technology, USA). After being washed with PBST for three times, the membranes were treated with horseradish peroxidase (HRP)-conjugated goat anti-rabbit IgG or goat anti-mouse IgG secondary antibody (Abbkine, USA). A chemiluminescence kit (Beyotime) was used to detect signals resulting from antibodies binding to membrane-bound proteins.

### SiRNA Transfection

SiRNAs targeting *eIF5A, eIF4E* and the negative control (NC) were synthesized by Gene Pharma (Shanghai, China) as described in Supplementary Table 2. CRL-2843-CD163 cells or PAMs were transfected with each indicated siRNA at a final concentration of 0.2 mM using Lipofectamine RNAiMAX (Invitrogen, USA) according to the manufacturer's instructions. Effects of transfected siRNAs after 24, 36, and 48 h were verified by RT-PCR using primers that are listed in Supplementary Table 1 and by WB analysis based on binding of mAb probes to eIF5A and eIF4E proteins. The rescue experiment was carried out according to a previous study (24), the siRNA targeting the 3′untranslated region (UTR) of *eIF5A* which was used in rescue experiment was synthesized by Gene Pharma (sequence is listed in Supplementary Table 2) and was used in the *eIF5A* knockdown experiment conducted in CRL-2843-CD163 cells. Cytotoxicity was assessed at 24, 36, and 48 h after siRNA transfection by adding MTS reagent to cells followed by incubation of cells at 37°C for 1 h. Absorbance was measured at 490 nm.

### Effect of *SiRNA-eIF5A* on PRRSV Infection

After CRL-2843-CD163 cells or PAMs were subjected to *eIF4E* and *eIF5A* knockdown with appropriate siRNAs (or NC control), the cells were inoculated with HP-PRRSV HN07-1 (MOI = 0.1) and harvested at 12 and 24 hpi for RT-PCR analysis. CRL-2843-CD163 cells or PAMs after *eIF5A* knockdown were inoculated with HP-PRRSV HN07-1 (MOI = 0.1) and harvested at 24 hpi for IFA and WB analyses. PAMs after *eIF5A* knockdown were infected with HP-PRRSV HN07-1 (MOI = 0.1) and harvested at 48 hpi for determination of the median tissue culture infective dose (TCID_50_), which was conducted as follows: MARC-145 cells were cultured in 96-well plates overnight in DMEM (Solarbio) containing 10% FBS. Next, the cells were inoculated with diluted PRRSV at a MOI of 0.1 followed by incubation at 37°C for 3 h. After the cells were washed, DMEM containing 2% FBS was added to each well-then viral yields were calculated based on TCID_50_ values determined at 48 hpi as per the Reed-Munch method (25).

### EIF5A Rescue Experiments

To determine whether recombinant eIF5A expression could reverse suppression of PRRSV infection due to *eIF5A* knockdown, we cloned *eIF5A* into a eukaryotic expression vector. Briefly, cDNA encoding eIF5A was amplified via PCR from full-length *eIF5A* cDNA (Gene ID: 100517970) using the primers listed in Supplementary Table 1. Next, the amplified PCR product was isolated and inserted into the 3^*^Flag-CMV-7.1 eukaryotic expression vector (Sigma) between the HindIII and XbaI sites. The recombinant 3^*^Flag-CMV-eIF5A eukaryotic expression vector was validated by the Shanghai Sangon Biotech Co. Ltd. (Shanghai, China). WB was used to confirm expression of 3^*^Flag-CMV-eIF5A protein in cells using anti-eIF5A mAb and anti-Flag mAb (Sigma). After siRNA targeting the 3′UTR of *eIF5A* was transfected into CRL-2843-CD163 cells, recombinant 3^*^Flag-CMV-eIF5A was transfected into the same cells then PRRSV propagation in the CRL-2843-CD163 cells was evaluated by RT-PCR, IFA, WB, and TCID_50_ assay. Cytotoxicity of 3^*^Flag-CMV-eIF5A after it was transfected into CRL-2843-CD163 cells (after *eIF5A* knockdown) was assessed at 24, 36, and 48 h post-transfection using the same method as described above.

### Statistical Analysis

Experimental data were expressed as the mean and ± standard deviation (SD) based on triplicate samples then data were analyzed via Student's *t*-test using GraphPad Prism software (v8.0). Statistical significance is indicated in the figures as ^*^, *P* < 0.05; ^**^, *P* < 0.01; ^***^, *P* < 0.001; ns, not significant.

## Results

### Propagation Kinetics of HP-PRRSV HN07-1 in PAMs

**Figure 1:**
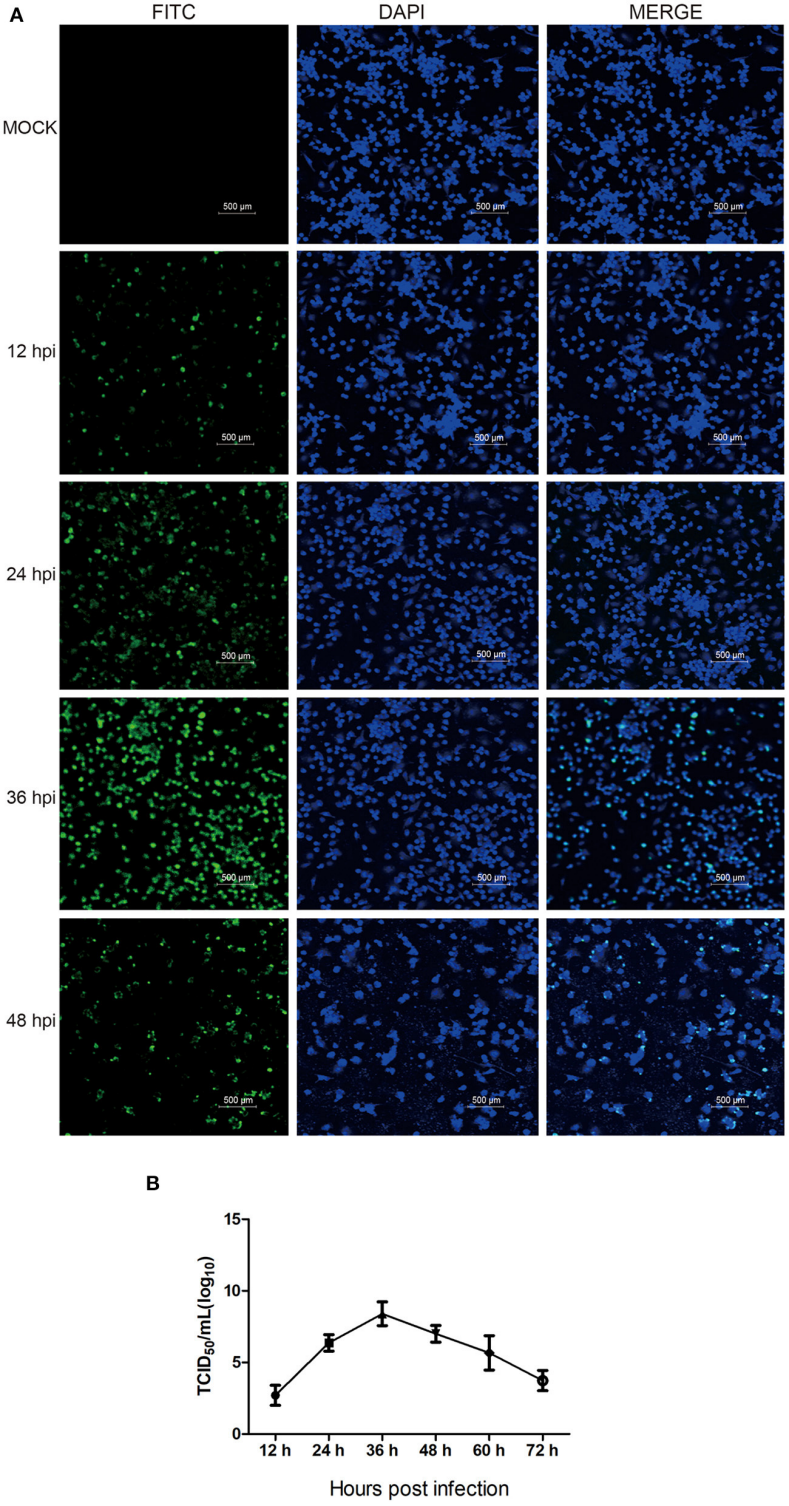
The propagation kinetics of HP-PRRSV HN07-1 in PAMs. **(A)** PAMs were infected with HP-PRRSV HN07-1 at MOI = 0.1 for 12, 24, 36, and 48 h or were mock-infected for 36 h as a control. Fluorescent images were recorded using an OLYMPUS IX 81 confocal microscope based on detection of virus using anti-PRRSV N protein antibody. **(B)** The one-step growth curve of HP-PRRSV HN07-1 in PAMs based on viral titers at 12, 24, 36, 48, 60, and 72 hpi.

First, PAMs were infected with HP-PRRSV HN07-1 at a MOI of 0.1. Next, to determine the appropriate time point for proteomic analysis, IFA was conducted of infected cells using a mAb probe specific for the viral N protein in order to determine the kinetics of HP-PRRSV HN07-1 infection in PAMs. As shown in Figure 1A, specific immunofluorescence became visible as early as 12 hpi that indicated that PRRSV propagation began at that time, with fluorescence significantly increasing after 24 hpi. At 36 hpi, fluorescence reached its maximum value then decreased at 48 hpi (Figure 1A). Based on these results, the one-step growth curve for HP-PRRSV HN07-1 in PAMs was plotted and it revealed that viral titers of HP-PRRSV HN07-1 reached a maximum of approximately 10^7.2^ TCID_50_/mL at 36 hpi then gradually declined (Figure 1B). Based on IFA and growth curve results, we chose the time point of 24 hpi (before the titer peaked at 36 hpi) for use in subsequent proteomic analyses.

### LC-MS/MS Detection

Using mock-infected and HP-PRRSV HN07-1-infected PAMs, label-free quantitative proteome analysis was performed on triplicate samples then the data for each sample were analyzed using Peaks 7.5 software tool in order to conduct database searching and quantitative analysis. The results revealed detection of total numbers of peptides (29,854, 26,947) and proteins (3794, 3558) in mock-infected and HP-PRRSV HN07-1-infected PAMs, respectively (Supplementary Tables 3–6). All proteomic data were deposited into the ProteomeXchange Consortium (http://proteomecentral.proteomexchange.org) iProX partner repository (26) using the dataset identifier PXD026209.

### Protein Quantification

**Figure 2:**
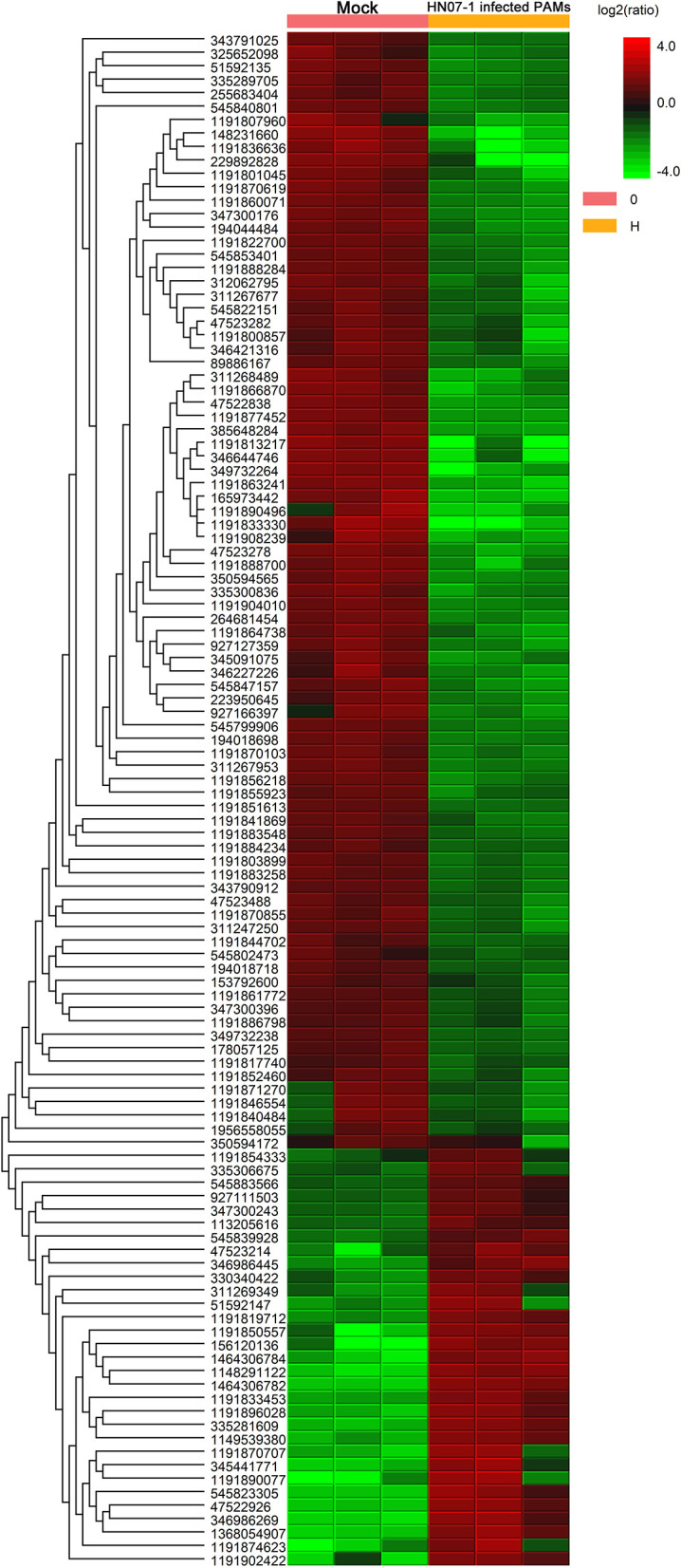
Clustering analysis of significantly differentially expressed proteins in HP-PRRSV HN07-1-infected PAMs. Unsupervised hierarchical clustering of differentially expressed (fold change ≥2 and *P* < 0.05) proteins in HP-PRRSV HN07-1-infected PAMs. The columns represent mock cells and HP-PRRSV-HN07-1-infected PAMs for three replicate samples, while rows represent different proteins. Up-regulated and down-regulated proteins are indicated by red and green colors, respectively, with color intensity reflecting magnitudes of protein expression level changes, as shown in the legend at the upper right. More information can be found in Supplementary Table 7.

Next, the quantitative function tool of PEAKS 7.5 (Bioinformatics Solutions Inc.) was used to examine clusters of significantly different proteins identified in HP-PRRSV HN07-1-infected PAMs at 24 hpi (>1.5-fold, *P* < 0.05). Ultimately, 269 significantly differentially expressed proteins were identified after HP-PRRSV HN07-1 infection (*P* < 0.05), of which 46 proteins were significantly up-regulated and 223 proteins were significantly down-regulated (Tables 1, 2). Importantly, for samples tested in triplicate, the heatmap indicated good repeatability (Figure 2). More information can be found in Supplementary Table 7.

**Table 1:** The significantly up-regulated proteins in HP-PRRSV HN07-1-infected PAMs identified by LC-MS/MS.

**No**	**Accession**	**Coverage** **(%)**	**Peptides**	**Unique**	**Group Profile**	**Description**
					**(Ratio)**	
1	gi|346421333	17	2	1	00:01.0	Non-histone chromosomal protein HMG-17
2	gi|324123893	9	3	3	00:01.0	Interferon-induced protein with tetratricopeptide repeats 3
3	gi|346986269	8	3	3	00:01.0	Interferon-induced protein with tetratricopeptide repeats 1
4	gi|545867461	8	5	4	00:01.0	Interferon-induced GTP-binding protein Mx1
5	gi|148231384	31	1	1	00:01.0	Thymosin beta-10
6	gi|311258597	1	1	1	00:01.0	Rootletin
7	gi|311250758	21	1	1	00:01.0	Interferon-induced transmembrane protein 1
8	gi|545812163	11	1	1	00:01.0	Histidine triad nucleotide-binding protein 1-like
9	gi|350529421	8	1	1	00:01.0	Mitochondrial ribosomal protein L49
10	gi|545884074	57	6	5	1.00:31.22	Thymosin beta-4
11	gi|545892076	15	3	3	1.00:11.28	Interferon-induced 17 kDa protein
12	gi|350583970	2	1	1	1.00:4.87	Keratin type II cytoskeletal 75
13	gi|345441792	2	1	1	1.00:3.31	Alcohol dehydrogenase 1C (class I) gamma polypeptide
14	gi|47522622	4	1	1	1.00:3.05	Galectin-9
15	gi|312283580	14	4	4	1.00:2.94	Superoxide dismutase [Mn] mitochondrial
16	gi|194036463	22	4	1	1.00:2.77	Ras-related protein Rap-1A
17	gi|545855071	2	1	1	1.00:2.77	Von Willebrand factor A domain-containing protein 8
18	gi|356460981	1	1	1	1.00:2.64	Probable ATP-dependent RNA helicase DDX58
19	gi|148235632	31	9	8	1.00:2.63	Interferon-induced GTP-binding protein Mx2
20	gi|350580091	5	1	1	1.00:2.61	Proteoglycan 3
21	gi|545811106	5	2	2	1.00:2.35	Sequestosome-1
22	gi|350582355	2	1	1	1.00:2.33	Exportin-1
23	gi|350539043	3	1	1	1.00:2.18	Phospholipid scramblase 1
24	gi|545808740	5	1	1	1.00:2.12	60S ribosomal protein L18a
25	gi|350586371	1	1	1	1.00:2.11	Coagulation factor XIII A chain
26	gi|297632416	14	2	2	1.00:1.88	Enhancer of rudimentary homolog
27	gi|148233143	3	1	1	1.00:1.81	Prolyl 4-hydroxylase subunit alpha-1 precursor
28	gi|545859997	3	1	1	1.00:1.80	Beta-arrestin-2
29	gi|72535204	4	1	1	1.00:1.79	Nicotinamide phosphoribosyl transferase
30	gi|194036682	3	1	1	1.00:1.77	Gamma-glutamyl hydrolase
31	gi|47523066	4	1	1	1.00:1.77	Caspase-3
32	gi|545821746	3	1	1	1.00:1.74	Tyrosine-protein kinase Lyn
33	gi|545856413	1	2	2	1.00:1.70	E3 ubiquitin-protein ligase RNF213-like partial
34	gi|47522754	1	1	1	1.00:1.68	Trifunctional enzyme subunit alpha mitochondrial
35	gi|237681310	24	3	3	1.00:1.66	Protein S100-A8
36	gi|545880266	3	1	1	1.00:1.65	RNA-binding protein Raly
37	gi|194034801	17	1	1	1.00:1.63	Normal mucosa of esophagus-specific gene 1 protein
38	gi|47523306	4	3	3	1.00:1.61	Signal transducer and activator of transcription 1
39	gi|350539097	10	1	1	1.00:1.61	Ubiquitin/ISG15-conjugating enzyme E2 L6
40	gi|347300243	9	5	5	1.00:1.57	Glutamate dehydrogenase 1 mitochondrial
41	gi|148225750	3	2	2	1.00:1.57	Heat shock protein 105 kDa
42	gi|350589740	11	1	1	1.00:1.56	60S ribosomal protein L21
43	gi|350580983	17	3	3	1.00:1.53	Receptor expression-enhancing protein 5
44	gi|50979305	1	1	1	1.00:1.52	Sialoadhesin precursor
45	gi|335279372	6	1	1	1.00:1.51	Myristoylated alanine-rich C-kinase substrate
46	gi|545887382	6	1	1	1.00:1.51	B-cell receptor-associated protein 31

**Table 2:** The significantly down-regulated proteins in HP-PRRSV HN07-1-infected PAMs identified by LC-MS/MS.

**No**	**Accession**	**Coverage (%)**	**Peptides**	**Unique**	**Group profile**	**Description**
					**(Ratio)**	
1	gi|264681454	8	2	2	1.00:0.67	S-adenosylmethionine synthase isoform type-2
2	gi|545801818	15	6	6	1.00:0.66	Cytosolic non-specific dipeptidase
3	gi|47522870	3	1	1	1.00:0.66	Serine/threonine-protein phosphatase 2A 65kDa regulatory subunit
4	gi|47522828	2	1	1	1.00:0.66	Transferrin receptor protein 1
5	gi|113205886	7	1	1	1.00:0.66	Nucleoside diphosphate kinase B
6	gi|350581449	9	3	1	1.00:0.66	Guanine nucleotide-binding protein G(I)/G(S)/G(T) subunit beta-2
7	gi|335282345	12	1	1	1.00:0.66	Mitochondrial import inner membrane translocase subunit Tim13
8	gi|347300400	6	1	1	1.00:0.66	Core histone macro-H2A.1
9	gi|311254887	3	1	1	1.00:0.66	Gasdermin-D
10	gi|346716314	18	2	2	1.00:0.65	Rho GDP dissociation inhibitor (GDI) beta
11	gi|545810927	5	2	2	1.00:0.65	Polypyrimidine tract-binding protein 1
12	gi|335283403	10	5	4	1.00:0.65	Lamin-B1
13	gi|350596594	3	1	1	1.00:0.65	Malate dehydrogenase cytoplasmic-like
14	gi|311258550	19	5	4	1.00:0.64	EF-hand domain-containing protein D2
15	gi|346644866	5	1	1	1.00:0.64	Coiled-coil-helix-coiled domain-containingprotein 3 mitochondrial
16	gi|148230268	25	8	8	1.00:0.64	Galectin-3
17	gi|545804280	6	1	1	1.00:0.64	Erythrocyte band 7 integral membrane protein
18	gi|350594565	13	5	5	1.00:0.64	Acid ceramidase-like
19	gi|545894790	28	4	4	1.00:0.64	Ribonuclease inhibitor partial
20	gi|545815108	2	1	1	1.00:0.64	Coronin-7
21	gi|311268187	7	1	1	1.00:0.64	Thioredoxin domain-containing protein 17 isoform 1
22	gi|545884463	7	4	4	1.00:0.64	Cytochrome b-245 heavy chain
23	gi|47522784	8	2	2	1.00:0.64	Fructose-1 6-bisphosphatase 1
24	gi|311252239	2	1	1	1.00:0.64	All-trans-retinol 13,14-reductase
25	gi|545877341	6	1	1	1.00:0.64	NAD(P) transhydrogenase mitochondrial-like
26	gi|545806957	9	31	31	1.00:0.63	AHNAK nucleoprotein
27	gi|335296249	14	2	2	1.00:0.63	Cysteine and glycine-rich protein 1-like isoform 3
28	gi|545894785	3	1	1	1.00:0.63	Heterogeneous nuclear ribonucleoprotein H2
29	gi|335296435	9	5	5	1.00:0.63	Cytoplasmic aconitate hydratase
30	gi|178056616	12	2	2	1.00:0.63	Rho-related GTP-binding protein RhoG
31	gi|545893961	5	1	1	1.00:0.63	Mannose-P-dolichol utilization defect 1 protein-like
32	gi|545894799	13	1	1	1.00:0.63	Nuclear transport factor 2-like partial
33	gi|172072665	5	4	2	1.00:0.63	Hexokinase-2
34	gi|343790912	22	4	2	1.00:0.63	Ras-related C3 botulinum toxin substrate 1
35	gi|350593002	10	1	1	1.00:0.63	Sideroflexin-3
36	gi|194043861	21	10	1	1.00:0.63	Tubulin alpha-1D chain
37	gi|229892818	21	2	2	1.00:0.62	Prothymosin alpha
38	gi|350587143	9	3	3	1.00:0.62	Legumain
39	gi|347300396	11	3	3	1.00:0.62	Histamine N-methyltransferase
40	gi|165973416	11	3	3	1.00:0.62	SLA class II histocompatibility antigen DQ haplotype C beta chain precursor
41	gi|545870539	1	1	1	1.00:0.62	Vinculin
42	gi|545830766	4	1	1	1.00:0.62	Heterogeneous nuclear ribonucleoprotein L
43	gi|298104128	22	1	1	1.00:0.62	Reactive oxygen species modulator
44	gi|545819344	5	1	1	1.00:0.61	40S ribosomal protein S7-like
45	gi|545867661	16	11	11	1.00:0.61	Integrin beta-2
46	gi|347300387	13	2	2	1.00:0.61	60S ribosomal protein L17
47	gi|194044822	7	2	1	1.00:0.61	Peroxiredoxin-4
48	gi|311259613	12	2	2	1.00:0.61	40S ribosomal proteinS5 isoform 1
49	gi|328550534	4	1	1	1.00:0.61	Electron transfer flavoprotein subunit beta
50	gi|311267953	21	3	3	1.00:0.61	60S ribosomal protein L23a-like
51	gi|346227212	4	2	2	1.00:0.61	ribosomal protein L3
52	gi|335292095	2	1	1	1.00:0.61	Mitogen-activated protein kinase 14
53	gi|545854442	12	2	2	1.00:0.60	High mobility group protein B1
54	gi|51592135	20	3	3	1.00:0.60	Cofilin-1
55	gi|114326183	8	1	1	1.00:0.60	ADP-ribosylation factor 4
56	gi|545848409	4	1	1	1.00:0.60	Acetyl-CoA acetyltransferase mitochondrial
57	gi|545894465	19	4	4	1.00:0.59	14-3-3 protein epsilon-like
58	gi|545887655	6	2	2	1.00:0.59	Glucose-6-phosphate 1-dehydrogenase partial
59	gi|545834779	2	1	1	1.00:0.59	Tyrosine-tRNA ligase cytoplasmic
60	gi|545876142	2	1	1	1.00:0.58	Leucine-rich repeat flightless-interacting protein 2
61	gi|335278864	6	2	1	1.00:0.58	L-lactate dehydrogenase A-like 6B-like
62	gi|335287489	2	1	1	1.00:0.58	Tubulin tyrosine ligase-like family member 12
63	gi|113205762	2	1	1	1.00:0.58	Granulins precursor
64	gi|311277265	3	1	1	1.00:0.58	55 kDa erythrocyte membrane protein
65	gi|194034199	4	1	1	1.00:0.58	Proteasome subunit alpha type-3
66	gi|545812127	12	1	1	1.00:0.58	Alpha-aminoadipic semialdehyde dehydrogenase
67	gi|545876461	1	1	1	1.00:0.58	Low quality protein: vigilin
68	gi|113205616	24	4	4	1.00:0.57	60S ribosomal protein L10
69	gi|545895140	24	2	2	1.00:0.57	Filamin-A partial
70	gi|47523694	7	2	2	1.00:0.57	calpain small subunit 1
71	gi|311275455	10	1	1	1.00:0.57	V-type proton ATPase subunit F
72	gi|545869404	3	1	1	1.00:0.57	RNA-binding protein EWS
73	gi|47523866	4	1	1	1.00:0.57	Aldose 1-epimerase
74	gi|545883733	3	1	1	1.00:0.56	Transcriptional activator protein Pur-beta-like partial
75	gi|359465556	1	1	1	1.00:0.56	Active breakpoint cluster region-related protein
76	gi|47522760	2	1	1	1.00:0.56	Long-chain 3-ketoacyl-CoA thiolase
77	gi|347658980	10	1	1	1.00:0.55	Ribosomal protein L36
78	gi|113205704	7	1	1	1.00:0.55	Proteasome subunit beta type-10
79	gi|346421419	2	1	1	1.00:0.55	Lysosome membrane protein 2 precursor
80	gi|48675931	16	2	1	1.00:0.55	40S ribosomal protein S17
81	gi|335281954	2	1	1	1.00:0.55	Rho GTPase-activating protein 1
82	gi|545830671	2	1	1	1.00:0.55	26S proteasome non-ATPase regulatory subunit 8
83	gi|346986249	6	1	1	1.00:0.55	Proteasome subunit beta type-1
84	gi|545873280	10	1	1	1.00:0.54	Activated RNA polymerase II transcriptional coactivator p15-like
85	gi|545839827	2	2	2	1.00:0.54	Exportin-5
86	gi|523580068	1	1	1	1.00:0.54	Chaperonin containing TCP1 subunit 5 (epsilon)
87	gi|545894159	3	1	1	1.00:0.54	Beta-galactosidase-like
88	gi|545847157	2	1	1	1.00:0.54	Integrin-linked protein kinase
89	gi|545871130	5	2	2	1.00:0.53	Lysosomal acid lipase/cholesteryl ester hydrolase
90	gi|349501107	36	2	1	1.00:0.53	Ribosomal protein large P2
91	gi|311275636	5	2	2	1.00:0.53	Septin-7
92	gi|311254975	3	1	1	1.00:0.53	NADH-cytochrome b5 reductase 3-like
93	gi|545858849	4	2	2	1.00:0.53	Retinoid-inducible serine carboxypeptidase
94	gi|346421386	18	1	1	1.00:0.53	ATP synthase subunit g mitochondrial
95	gi|113205690	5	2	1	1.00:0.53	Protein phosphatase 1 catalytic subunit alpha isoform
96	gi|311261988	14	6	6	1.00:0.52	Phosphoglucomutase-2
97	gi|350584895	3	1	1	1.00:0.52	Protein VAC14 homolog partial
98	gi|545894912	6	2	2	1.00:0.52	Apolipoprotein B receptor partial
99	gi|545860680	2	1	1	1.00:0.52	Transient receptor potential cation channel subfamily V member 2
100	gi|350582454	1	1	1	1.00:0.52	Leucine-rich PPR motif-containing protein mitochondrial
101	gi|346986437	9	2	2	1.00:0.52	Family with sequence similarity 49 member B
102	gi|356582297	15	3	1	1.00:0.52	ADP-ribosylation factor-like 8B
103	gi|545828227	12	5	5	1.00:0.51	Leukotriene A-4 hydrolase
104	gi|47523292	15	2	2	1.00:0.51	CD74 antigen
105	gi|350595800	9	1	1	1.00:0.51	SH3 domain-binding glutamic acid-rich-like protein-like
106	gi|340007404	13	8	7	1.00:0.51	Alpha-actinin-1
107	gi|194040450	9	2	2	1.00:0.51	Lactoylglutathione lyase isoform 1
108	gi|545883591	7	1	1	1.00:0.51	Chromobox protein homolog 3
109	gi|335282824	3	1	1	1.00:0.51	IlvB (bacterial acetolactate synthase)-like
110	gi|335281298	14	6	1	1.00:0.50	Tubulin beta-4B chain
111	gi|324021713	20	6	6	1.00:0.50	Ribosomal protein S4
112	gi|545825344	10	1	1	1.00:0.50	UPF0160 protein MYG1 mitochondrial-like
113	gi|329663948	6	2	1	1.00:0.50	Ras GTPase-activating protein-binding protein 1
114	gi|47522648	2	1	1	1.00:0.50	Beta-hexosaminidase subunit beta precursor
115	gi|305855130	2	2	2	1.00:0.50	Valyl-tRNA synthetase
116	gi|178057125	4	1	1	1.00:0.50	Cathepsin Z precursor
117	gi|545825997	14	3	3	1.00:0.49	Proliferation-associated protein 2G4-like
118	gi|311255664	3	3	3	1.00:0.48	Extended synaptotagmin-1
119	gi|269914120	11	2	2	1.00:0.48	Lysozyme C-3 precursor
120	gi|545827028	6	2	2	1.00:0.48	Branched-chain-amino-acid aminotransferase cytosolic
121	gi|311274648	7	1	1	1.00:0.48	Sulfiredoxin-1-like
122	gi|348605266	7	1	1	1.00:0.48	Ribosomal protein S13
123	gi|55926217	32	3	3	1.00:0.47	Cytochrome c oxidase subunit 5B mitochondrial precursor
124	gi|545862394	8	2	1	1.00:0.47	Guanine nucleotide-binding protein G(i) subunit alpha-2
125	gi|335286747	12	1	1	1.00:0.47	Hepatoma-derived growth factor
126	gi|47522692	3	1	1	1.00:0.47	Long-chain specific acyl-CoA dehydrogenase mitochondrial precursor
127	gi|83921637	3	1	1	1.00:0.47	Matrix metalloproteinase-9
128	gi|223950631	42	2	2	1.00:0.46	Guanine nucleotide-binding protein G(I)/G(S)/G(O) subunit gamma-12
129	gi|47522940	3	1	1	1.00:0.46	Dihydrolipoyl dehydrogenase mitochondrial precursor
130	gi|345110604	4	1	1	1.00:0.46	LIM and senescent cell antigen-like domains 1
131	gi|335304552	8	3	3	1.00:0.45	Acetyl-coenzyme A synthetase 2-like mitochondrial
132	gi|545838059	4	1	1	1.00:0.45	BOLA class I histocompatibility antigen alpha chain BL3-7
133	gi|147899011	13	1	1	1.00:0.45	40S ribosomal protein S26
134	gi|335309772	8	1	1	1.00:0.45	Acyl-coenzyme A thioesterase 2 mitochondrial-like partial
135	gi|47522782	10	1	1	1.00:0.44	Beta-2-microglobulin precursor
136	gi|350593430	2	1	1	1.00:0.44	Glutathione reductase mitochondrial isoform 1
137	gi|545894793	11	1	1	1.00:0.44	Vesicle-associated membrane protein 3-like
138	gi|154147607	2	1	1	1.00:0.44	Calpain-2 catalytic subunit
139	gi|116175251	8	1	1	1.00:0.43	Macrophage migration inhibitory factor
140	gi|356460899	12	5	5	1.00:0.43	Catalase
141	gi|89886167	36	5	5	1.00:0.42	Fatty acid-binding protein epidermal NADH dehydrogenase
142	gi|148225172	15	1	1	1.00:0.42	[Ubiquinone] 1 alpha subcomplex subunit 4
143	gi|545803550	5	1	1	1.00:0.42	Stomatin-like protein 2 mitochondrial
144	gi|47523720	12	7	7	1.00:0.42	Glucose-6-phosphate isomerase
145	gi|47523764	52	7	2	1.00:0.40	Peptidyl-prolyl cis-trans isomerase A
146	gi|335289972	16	2	2	1.00:0.40	Neutral amino acid transporter B(0)-like
147	gi|311254226	13	1	1	1.00:0.40	40S ribosomal protein S27
148	gi|545859904	6	3	1	1.00:0.40	Beta-enolase
149	gi|47523126	3	1	1	1.00:0.40	Ficolin-2 precursor
150	gi|350597193	12	3	3	1.00:0.40	Peptidyl-prolyl cis-trans isomerase B
151	gi|545877091	2	1	1	1.00:0.40	Disabled homolog 2
152	gi|350584410	2	1	1	1.00:0.40	Lysophospholipid acyltransferase 5
153	gi|194035847	7	1	1	1.00:0.39	Astrocytic phosphoprotein PEA-15
154	gi|311262781	8	1	1	1.00:0.39	Ragulator complex protein LAMTOR3 isoform 1
155	gi|311247250	35	2	2	1.00:0.38	barrier-to-autointegration factor-like
156	gi|335300836	5	1	1	1.00:0.38	ES1 protein homolog mitochondrial
157	gi|545856802	5	1	1	1.00:0.38	CMRF35-like molecule 1
158	gi|47523668	8	1	1	1.00:0.38	Microsomal glutathione S-transferase 1
159	gi|350590733	1	2	2	1.00:0.37	Pre-mRNA-processing-splicing factor 8 partial
160	gi|363814526	1	1	1	1.00:0.37	Macrophage mannose receptor 1 precursor
161	gi|335287593	19	3	1	1.00:0.37	Ras-related C3 botulinum toxin substrate 2
162	gi|545844580	3	1	1	1.00:0.37	RNA-binding protein 47
163	gi|157279731	0	1	1	1.00:0.37	Myosin-1
164	gi|545831942	2	1	1	1.00:0.37	Glycogen [starch] synthase muscle
165	gi|47523548	24	2	2	1.00:0.36	Glutaredoxin-1
166	gi|311260951	3	1	1	1.00:0.36	Dehydrogenase/reductase SDR family member 1
167	gi|545875118	1	1	1	1.00:0.35	Splicing factor 3B subunit 1
168	gi|545858898	25	1	1	1.00:0.34	Dynein light chain 2 cytoplasmic-like
169	gi|350578528	13	2	2	1.00:0.34	Peptidyl-prolyl cis-trans isomerase B partial
170	gi|47522836	19	2	2	1.00:0.34	Osteoclast-stimulating factor 1
171	gi|545856859	14	1	1	1.00:0.34	60S ribosomal protein L38
172	gi|545881923	7	2	2	1.00:0.33	GTPase IMAP family member 4
173	gi|194018718	25	3	3	1.00:0.33	40S ribosomal protein S20
174	gi|307746897	15	2	2	1.00:0.33	Protein-L-isoaspartate(D-aspartate) O-methyltransferase
175	gi|311258112	3	1	1	1.00:0.33	Myeloid-associated differentiation marker
176	gi|47523692	41	5	5	1.00:0.32	Thioredoxin
177	gi|311270662	22	2	2	1.00:0.32	Phosphatidylethanolamine-binding protein 1
178	gi|47522916	4	1	1	1.00:0.32	Glutathione S-transferase omega-1
179	gi|545808462	10	2	2	1.00:0.30	ADP-ribosylation factor 1
180	gi|545855599	0	1	1	1.00:0.30	Dedicator of cytokinesis protein 9
181	gi|350582111	1	1	1	1.00:0.30	Eukaryotic translation initiation factor 5B
182	gi|343478222	9	1	1	1.00:0.29	Eukaryotic translation initiation factor 4H
183	gi|545804271	7	1	1	1.00:0.29	Ras-related protein Rab-14
184	gi|311245496	23	1	1	1.00:0.26	Guanine nucleotide-binding protein G(I)/G(S)/G(O) subunit
185	gi|346227226	23	3	3	1.00:0.24	40S ribosomal protein S11
186	gi|55742824	4	1	1	1.00:0.24	Spliceosome RNA helicase DDX39B
187	gi|350582722	20	4	2	1.00:0.22	14-3-3 protein theta
188	gi|545803693	7	1	1	1.00:0.22	Clathrin light chain A
189	gi|545892437	11	2	2	1.00:0.21	CD9 antigen-like
190	gi|545817119	8	2	2	1.00:0.20	Pleckstrin
191	gi|317054710	4	1	1	1.00:0.20	Cytochrome c oxidase subunit II (mitochondrion)
192	gi|545834956	0	1	1	1.00:0.20	Microtubule-actin cross-linking factor 1
193	gi|194018698	21	2	2	1.00:0.20	Cytochrome c
194	gi|343478210	12	2	2	1.00:0.20	Syntaxin 12
195	gi|545894926	9	1	1	1.00:0.20	Vacuolar protein sorting-associated protein 35-like partial
196	gi|47523666	21	4	4	1.00:0.19	Proteasome activator complex subunit 1
197	gi|545839153	6	1	1	1.00:0.19	60S ribosomal protein L10a
198	gi|346644790	22	1	1	1.00:0.19	Eukaryotic translation initiation factor 4E-binding protein 1
199	gi|545870732	5	1	1	1.00:0.17	Uncharacterized protein
200	gi|545825732	5	1	1	1.00:0.17	CD63 antigen
201	gi|47523278	11	1	1	1.00:0.16	Resistin precursor
202	gi|194041813	3	1	1	1.00:0.16	Phosphatidylinositol 4-kinase type 2-alpha
203	gi|311250199	2	1	1	1.00:0.15	Heterogeneous nuclear ribonucleoprotein A0
204	gi|47522778	2	1	1	1.00:0.15	Scavenger receptor cysteine-rich type 1 protein M130 precursor
205	gi|237681312	43	4	4	1.00:0.14	Protein S100-A12
206	gi|545846943	6	1	1	1.00:0.13	Heterogeneous nuclear ribonucleoprotein D-like
207	gi|312233368	15	1	1	1.00:0.12	ATP synthase F0 subunit 8 (mitochondrion)
208	gi|311268292	19	3	3	1.00:0.10	Eukaryotic translation initiation factor 5A
209	gi|545803799	12	3	2	1.00:0.07	Acidic leucine-rich nuclear phosphoprotein 32 family member
210	gi|545806769	5	1	1	1.00:0.06	Reticulon-3
211	gi|48374063	6	3	1	1.00:0.06	Desmin
212	gi|147903958	21	1	1	1.00:0.04	Cystatin-B
213	gi|545851826	10	1	1	1.00:0.04	Acyl carrier protein mitochondrial
214	gi|47523802	8	1	1	1.00:0	Translationally-controlled tumor protein
215	gi|213021237	7	1	1	1.00:0	DNA-(apurinic or apyrimidinic site) lyase
216	gi|311252670	22	4	1	1.00:0	Calmodulin-like
217	gi|350579215	41	6	1	1.00:0	Peptidyl-prolyl cis-trans isomerase A-like
218	gi|545815969	0	1	1	1.00:0	E3 SUMO-protein ligase RanBP2
219	gi|48675935	6	1	1	1.00:0	60S ribosomal protein L32
220	gi|335281875	4	1	1	1.00:0	Proteoglycan 3-like
221	gi|342349338	1	1	1	1.00:0	ElaC homolog 2
222	gi|545894997	18	1	1	1.00:0	SH3 domain-binding glutamic acid-rich-like protein 3 partial
223	gi|47523608	5	1	1	1.00:0	Cytochrome b-245 light chain

### Bioinformatics Analysis Based on ClueGo

**Figure 3:**
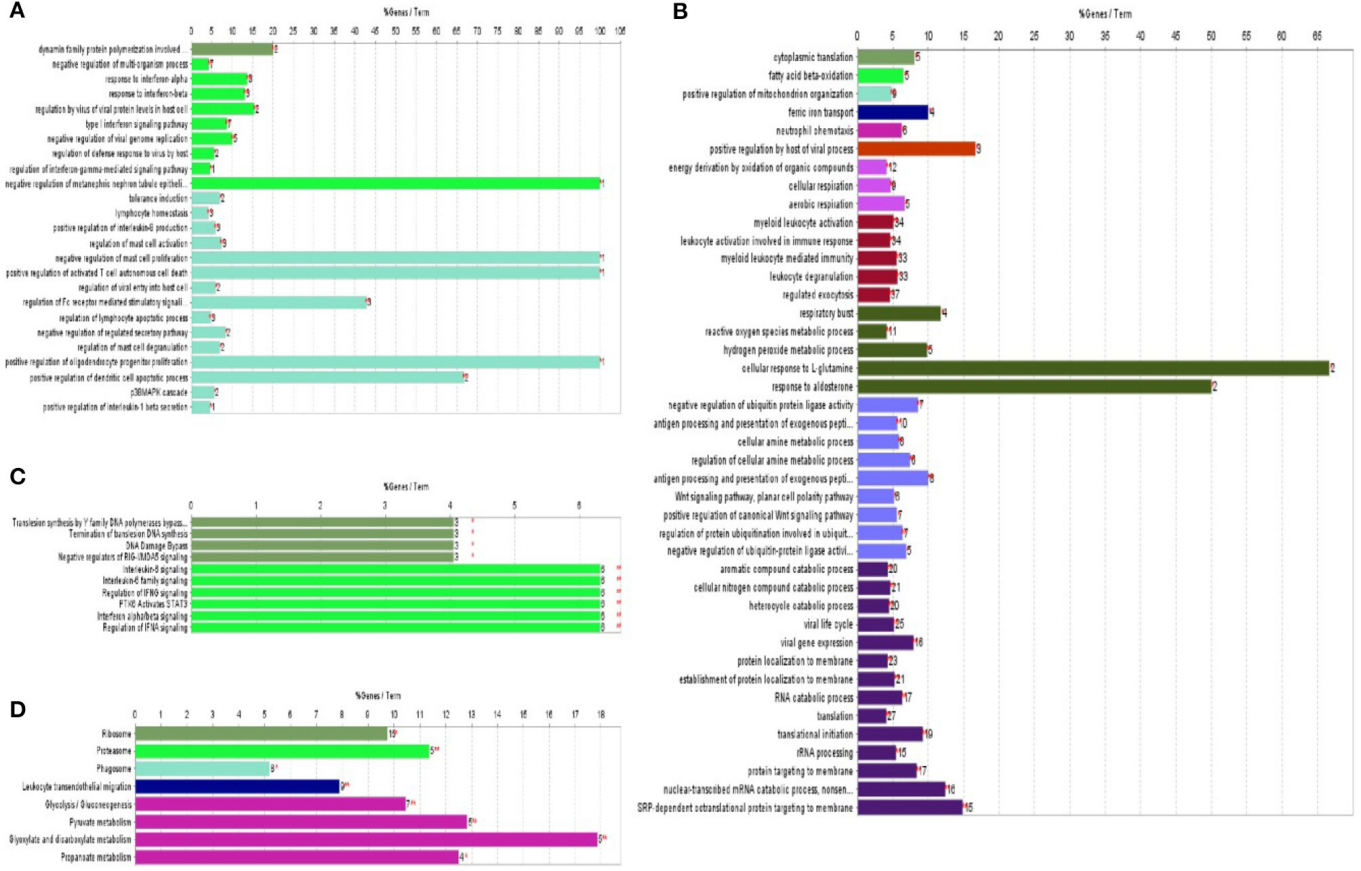
ClueGo was applied to enrich the biological process and KEGG pathways of significantly differentially expressed proteins identified in HP-PRRSV HN07-1-infected PAMs. **(A)** Biological process enrichment terms of significantly up-regulated proteins. **(B)** Biological process enrichment terms of significantly down-regulated proteins. **(C)** KEGG pathway enrichment terms of significantly up-regulated proteins. **(D)** KEGG pathway enrichment terms of significantly down-regulated proteins. The same color indicates similar functional categories. *P < 0.05, **P < 0.01. More information can be found in Supplementary Tables 8–11.

ClueGo V2.1.7 was used to generate functionally grouped annotation networks so that we could functionally categorize significantly differentially expressed proteins associated with HP-PRRSV HN07-1 infection. As shown in Figure 3A, up-regulated proteins were mainly associated with functional terms such as response to interferon (IFN)-α (*P* = 1.03 × 10^−5^), IFN-β (*P* = 1.18 × 10^−5^), positive regulation of interleukin (IL)-8 production (*P* = 1.34 × 10^−4^), positive regulation of IL-1β production (*P* = 4.22 × 10^−2^), and regulation of Fc receptor-mediated stimulatory signaling pathway (*P* = 2.38 × 10^−7^). Down-regulated proteins were mainly associated with functional terms such as cytoplasmic translation (*P* = 1.78 × 10^−04^), translation (*P* = 1.02 × 10^−11^), translational initiation (*P* = 1.07 × 10^−14^), and antigen processing and presentation of exogenous peptide antigen (*P* = 2.8 × 10^−06^) (Figure 3B). Additional data are presented in Supplementary Tables 8, 9.

ClueGo V 2.1.7 was next used to conduct KEGG pathway analysis to explore potential functional networks of differentially expressed proteins. Up-regulated proteins were mainly enriched for KEGG pathway terms such as IL-6 signaling (*P* = 4.46 × 10^−06^), regulation of IFN signaling (*P* = 4.45757089317539 × 10^−06^), and termination of translesion DNA synthesis (*P* = 1.8771367 × 10^−02^) (Figure 3C). Down-regulated proteins were mainly enriched for the KEGG term ribosome (*P* = 7.5 × 10^−08^) (Figure 3D). Additional data are shown in Supplementary Tables 10, 11. Taken together, bioinformatic analysis results revealed that after HP-PRRSV HN07-1 infection, host innate immunity-related proteins and pathways were almost all up-regulated, while translation-related proteins and processes were dramatically down-regulated.

### PPI Network Analysis

**Figure 4:**
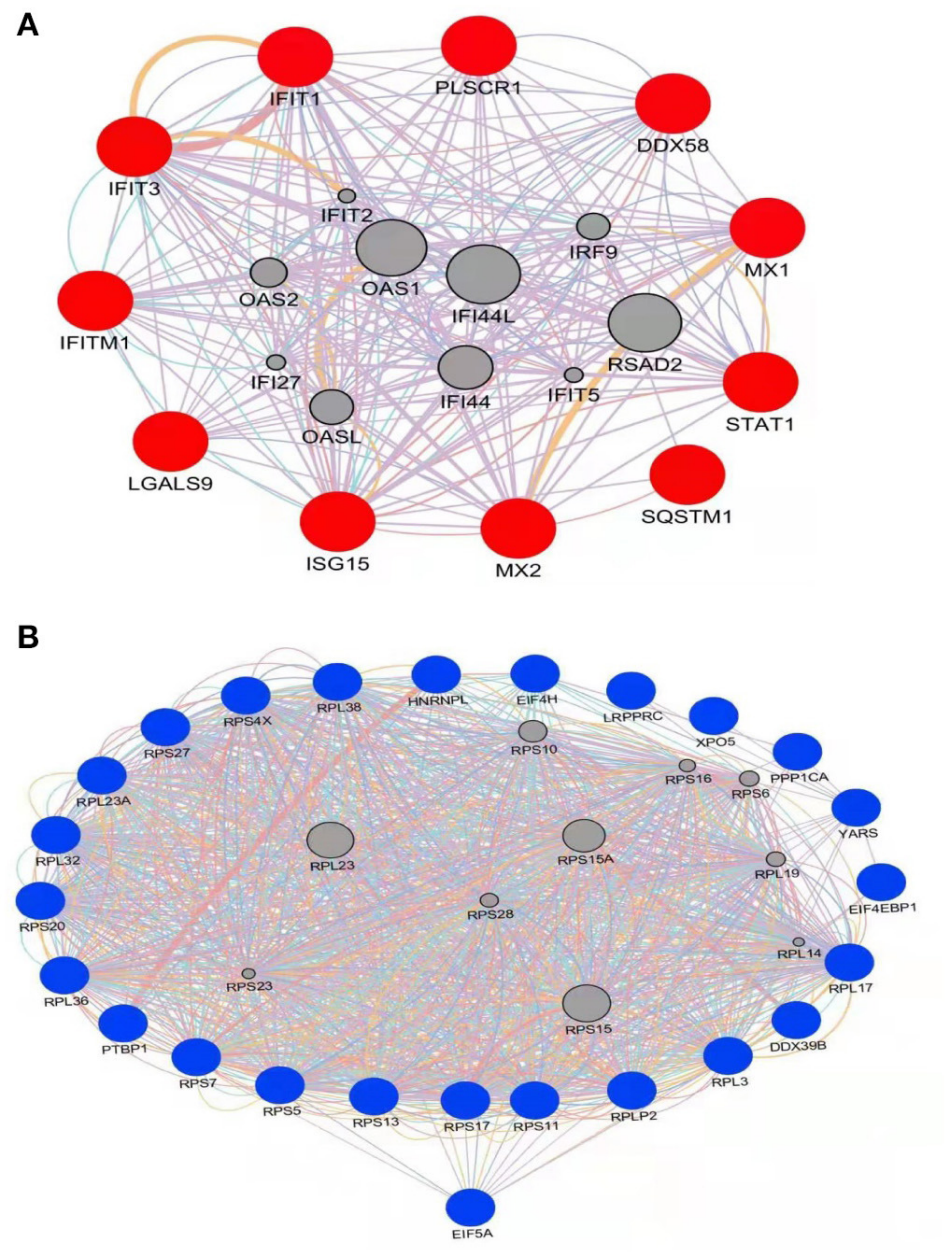
PPI network with significantly differentially expressed proteins in HP-PRRSV infected-PAMs using the GeneMANIA tool. **(A)** Significantly up-regulated proteins are shown in red; **(B)** Significantly down-regulated proteins are shown in blue. Edge: interaction between two different proteins.

To gain additional insights into possible functional interactions among the identified proteins, PPI networks were constructed using GeneMANIA (23) based on a large set of applicable association data pertaining to protein and genetic interactions (Figure 4). Due to the fact that the pig genome database is under-annotated, gene identifications for identified significantly differentially regulated proteins (listed in Tables 1, 2) were converted to human protein GI numbers. Next, predicted and integrated known PPI data sets from the *Homo sapiens* genome were input into GeneMANIA. Thereafter, GO categories were enriched for the input dataset using a false discovery rate (FDR)-corrected hypergeometric test.The results were compared to the background set of GO annotations for the entire *Homo sapiens* genome, with predicted, genetic, and physical interactions enabled during creation of the networks. The top 20 related genes and 20 attributes were displayed using GO biological process-based weighting, then Cytoscape was used to depict the networks. Figure 4A shows the interaction network for up-regulated proteins in HP-PRRSV HN07-1-infected PAMs that highlights interactions between host innate immunity and IFN I-related signaling pathway proteins. Figure 4B shows the interaction network for down-regulated proteins in HP-PRRSV HN07-1-infected PAMs that highlights the importance of interactions involving host proteins related to protein translation.

### RT-PCR and WB Analyses of Up-Regulated IFN I-Mediated Signaling Pathways

**Figure 5:**
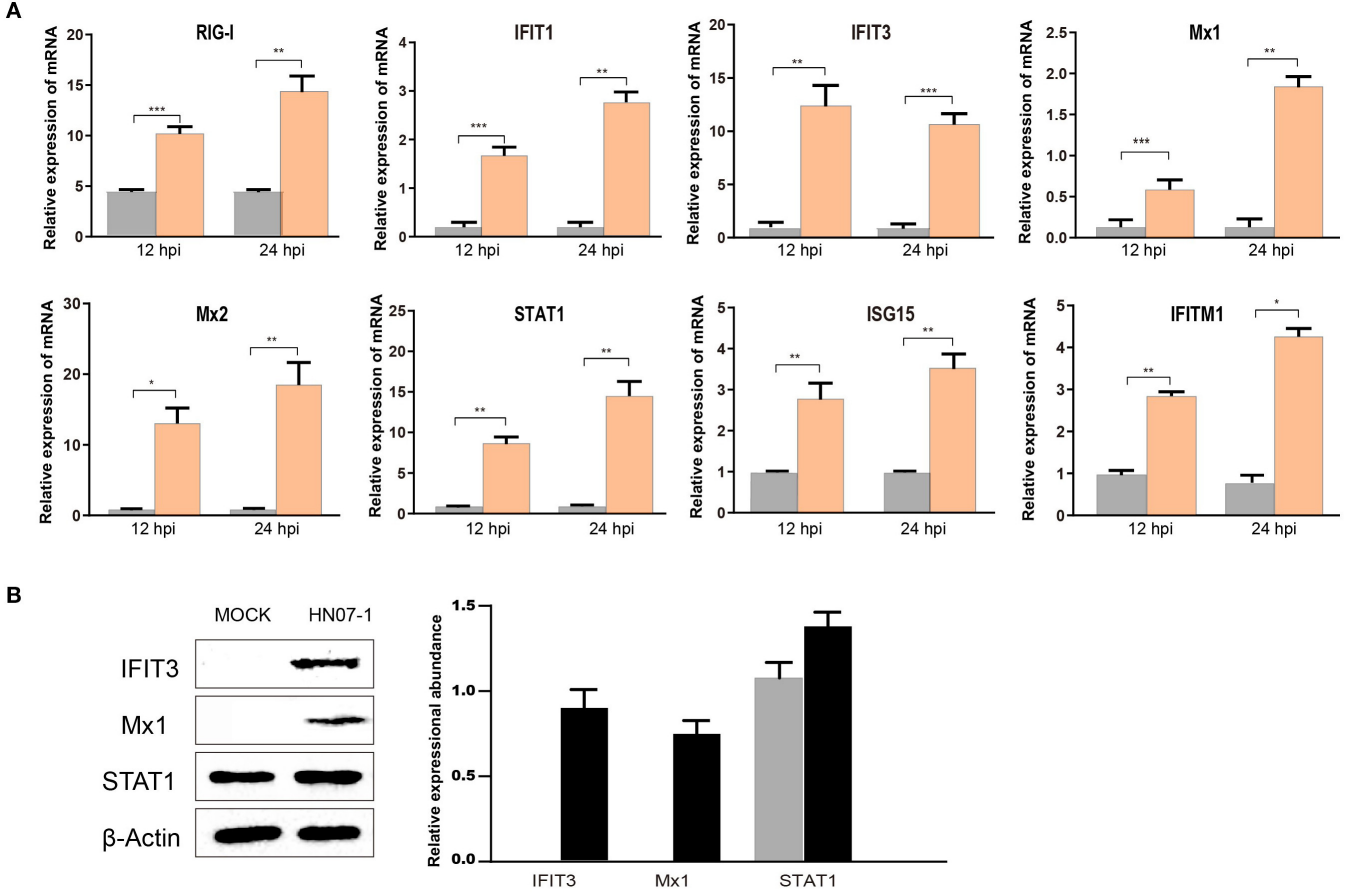
Confirmation of up-regulated proteins using RT-PCR and WB analyses. **(A)** PAMs were infected with HP-PRRSV HN07-1 at MOI = 0.1 or mock-infected. Samples were collected at 24 hpi for RT-PCR detection for analysis of relative mRNA abundances of RIG-I, IFIT1, IFIT3, Mx1, Mx2, ISG15, STAT1, and IFITM1. **(B)** PAMs were infected with HP-PRRSV at MOI = 0.1 or mock-infected. Samples were collected at 24 hpi for WB to analyze protein expression levels of STAT1, IFIT3, Mx1, and β-Actin. *P < 0.05; **P < 0.01; ***P < 0.001; ns, not significant.

Notably, proteins in IFN I-mediated signaling pathways were found to be up-regulated in our study. Given the importance of IFN-related proteins in host antiviral responses, we conducted RT-PCR to measure mRNA-level expression of genes that encode IFN-induced proteins, such as retinoic acid-inducible gene I (RIG-I), IFIT1, IFIT3, Mx1, Mx2, STAT1, interferon-stimulated gene 15 (ISG15), and interferon-induced transmembrane protein 1 (IFITM1) in HP-PRRSV HN07-1-infected or mock-infected PAMs. Intriguingly, abundances of mRNAs corresponding to these proteins were found to be significantly increased after HP-PRRSV HN07-1 infection (Figure 5A), with increased levels of Mx1, IFIT3, and STAT1 proteins confirmed by WB analysis (Figure 5B). Importantly, these results aligned with our MS results.

### Decreased EIF5A Abundance After HP-PRRSV HN07-1 Infection

**Figure 6:**
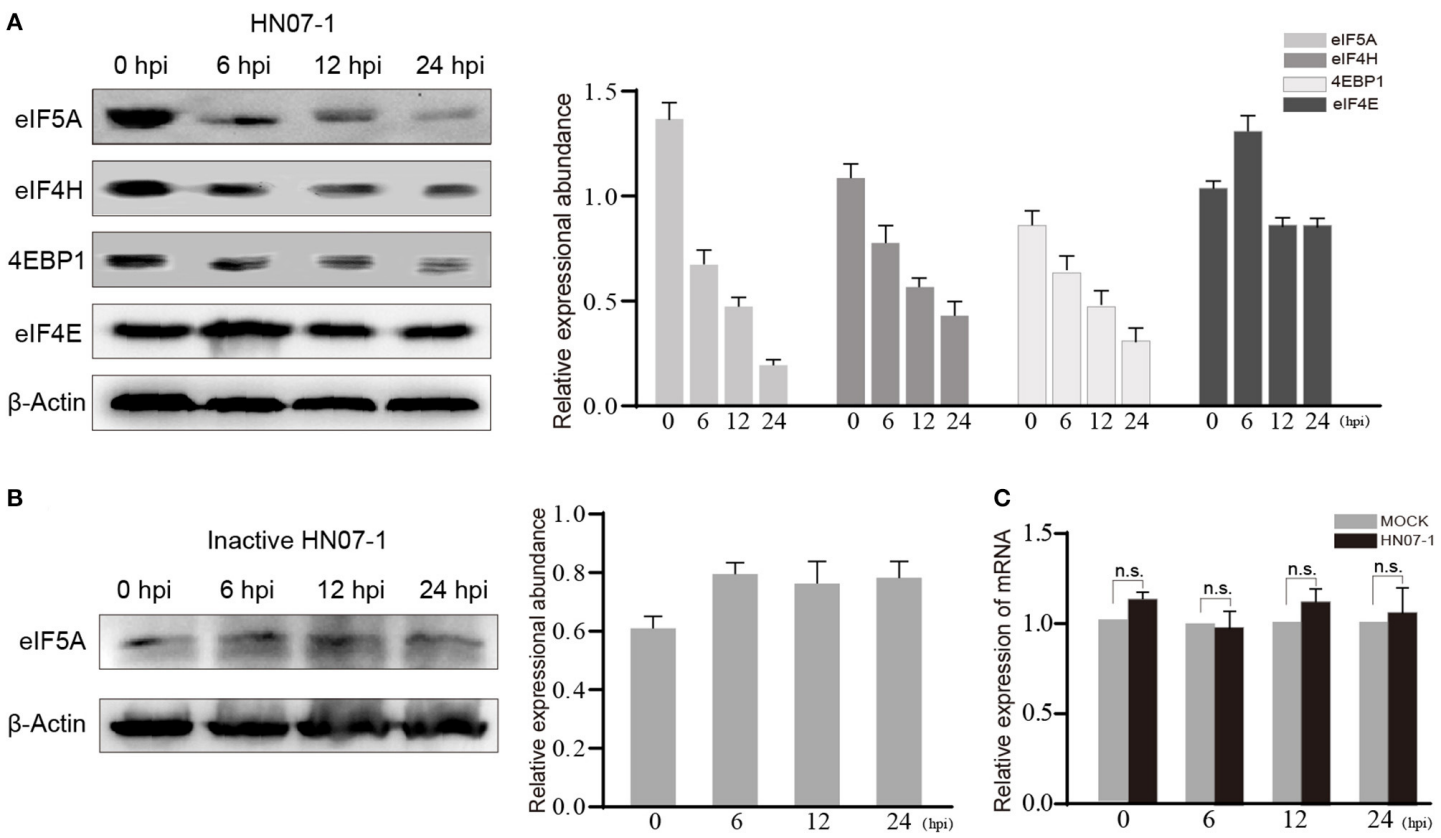
The protein level of eIF5A was decreased after HP-PRRSV HN07-1 infection. **(A)** PAMs were infected with HP-PRRSV HN07-1 at MOI = 0.1 or mock-infected. The cells were collected at 0, 6, 12, and 24 hpi then total proteins of cells were analyzed via WB to measure protein levels of eIF5A, 4EBP1, eIF4H, and eIF4E. **(B)** PAMs were treated with UV-inactivated PRRSV then the cells were harvested at 0, 6, 12, and 24 hpi, then total proteins from cells were analyzed via WB to measure protein levels of eIF5A. **(C)** PAMs were infected with HP-PRRSV HN07-1 at MOI = 0.1 or mock-infected. The cells were collected at 0, 6, 12, and 24 hpi then total RNA preparations from the cells were analyzed via RT-PCR to detect transcription-level expression of eIF5A.

Translation and translational initiation related proteins were found down-regulated in the current study. The protein levels of eIF5A, eIF4E, eIF4E-binding protein 1(4EBP1) and eIF4H in HP-PRRSV HN07-1-infected PAMs at 0, 6, 12 and 24 hpi were determined by WB. The protein levels of eIF5A, 4EBP1, and eIF4H were significantly decreased after 6 hpi, whereas that of eIF4E was not significantly altered (Figure 6A). Considering that eIF5A expression was down-regulated most significantly after PRRSV HN07-1 infection along with the fact that this protein has not been extensively researched in the field of virology, we chose eIF5A as a target for further study and used eIF4E as a control. Next, we tested eIF5A dynamics after PAMs were exposed to UV-inactivated PRRSV, with the results revealing that the eIF5A level remained stable in PAMs treated with UV-inactivated virions (Figure 6B). Interestingly, transcription-level expression of *eIF5A* in HP-PRRSV HN07-1-infected PAMs remained almost unchanged at all time points (0, 6, 12, and 24 hpi) (Figure 6C).

### Effect of *EIF5A* Knockdown on PRRSV Propagation *in vitro*

**Figure 7:**
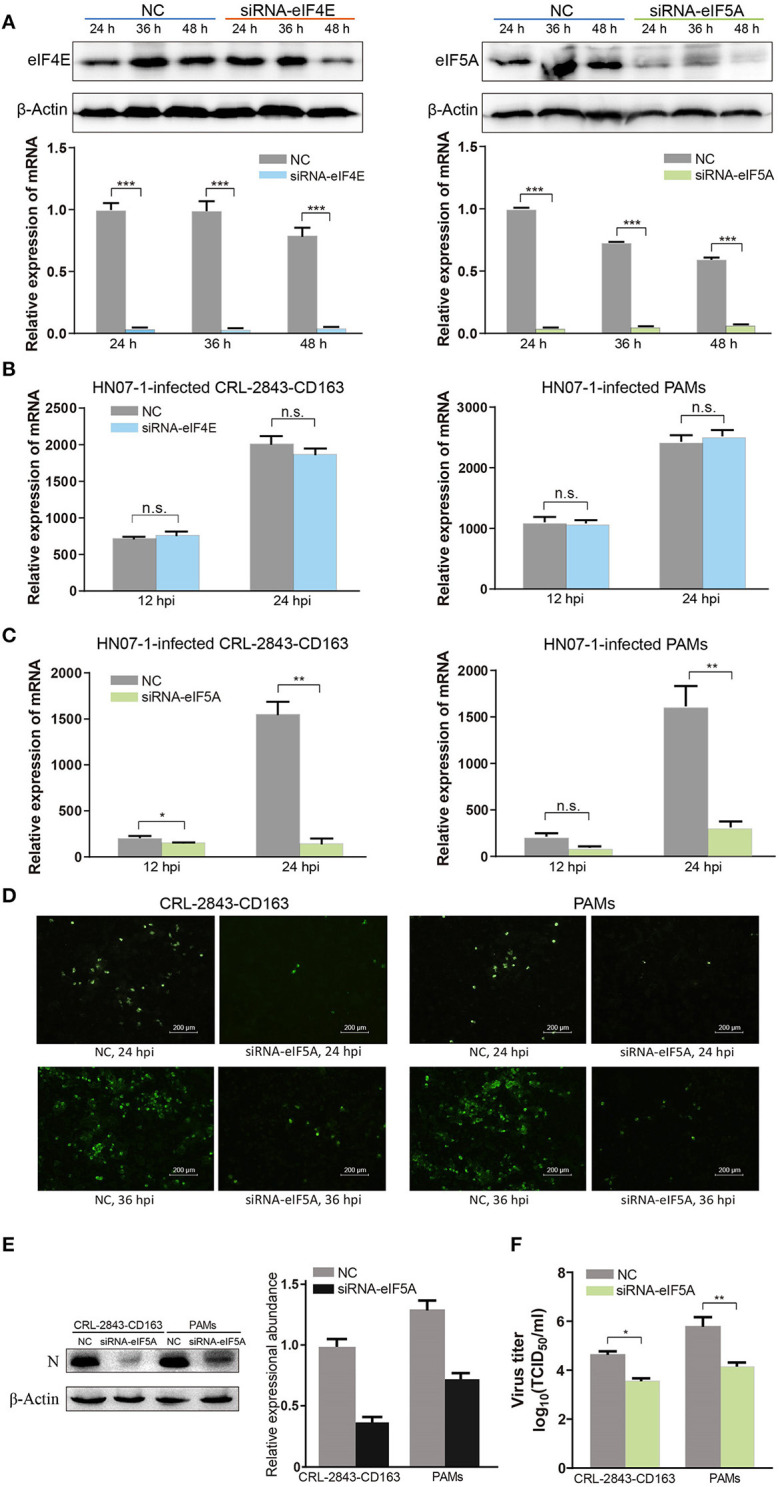
EIF5A is important for PRRSV infection *in vitro*. **(A)** *SiRNA-eIF4E* and *siRNA-eIF5A* were transfected into CRL-2843-CD163 cells for 24, 36, or 48 h, with NC transfected as the control. The knockdown effect was validated by RT-PCR and WB. **(B)** The *eIF4E* knockdown CRL-2843-CD163 cells or PAMs were inoculated with HP-PRRSV HN07-1 (MOI = 0.1) and harvested at 12 and 24 hpi for RT-PCR analysis. **(C)** The *eIF5A* knockdown CRL-2843-CD163 cells or PAMs were inoculated with HP-PRRSV HN07-1 (MOI = 0.1) and harvested at 12 and 24 hpi for RT-PCR analysis. **(D)** HP-PRRSV HN07-1 (MOI = 0.1) was used to inoculate *eIF5A* knockdown CRL-2843-CD163 cells or PAMs and harvested at 24 hpi for IFA analysis with anti-PRRSV N protein antibody. **(E)** HP-PRRSV HN07-1 (MOI = 0.1) was used to inoculate *eIF5A* knockdown CRL-2843-CD163 cells or PAMs then cells were harvested at 24 hpi for WB analysis with anti-PRRSV N protein antibody. **(F)** HP-PRRSV HN07-1 (MOI = 0.1) was used to inoculate *eIF5A* knockdown CRL-2843-CD163 cells or PAMs then cells were harvested at 48 hpi. Viral yields were determined based on TCID_50_ values in MARC-145 cells. Each experiment was carried out three times independently and yielded consistent findings. *P < 0.05; **P < 0.01; ***P < 0.001; ns, not significant.

We next studied the biological significance of eIF5A on PRRSV infection using the CRL-2843-CD163 cell line and PAMs. CRL-2843-CD163, a cell line that stably expresses CD163, was obtained by transfection of immortalized PAMs (CRL-2843) with DNA encoding the host PRRSV receptor CD163; thus, these cells can be readily infected with PRRSV. As shown in Figure 7A, RT-RCR and WB were carried out to verify effects of *eIF4E* and *eIF5A* knockdown in CRL-2843-CD163 cells. At 24 h after transfection, RT-PCR results indicated that transcriptional expression levels of *eIF5A* and *eIF4E* were dramatically reduced. WB analysis indicated that expression levels of eIF5A and eIF4E proteins were markedly decreased at 48 h after transfection (Figure 7A).

Next, CRL-2843-CD163 cells and PAMs were transfected with small interfering RNA (siRNA)-eIF4E and siRNA-eIF5A for 48 h, then were infected with HP-PRRSV HN07-1 and harvested at 24 hpi. Thereafter, RT-PCR was applied to verify the effects of transfected siRNAs on HP-PRRSV HN07-1 replication *in vitro*. The results showed that PRRSV replication in cells with knockdown of *eIF4E* expression (both CRL-2843-CD163 cells and PAMs) was not significantly different from that of the NC group (Figure 7B). By contrast, viral propagation in cells with knockdown of *eIF5A* expression was significantly inhibited (Figure 7C). In addition, expression of PRRSV N protein, as measured using IFA, was considerably suppressed in cells that were knockdown of *eIF5A* expression (both CRL-2843-CD163 cells and PAMs) (Figure 7D). Moreover, WB results revealed that N protein expression was dramatically reduced in cells after *eIF5A* knockdown (both CRL-2843-CD163 cells and PAMs) (Figure 7E). Furthermore, TCID_50_ values were much lower in cells after *eIF5A* knockdown (both CRL-2843-CD163 cells and PAMs) than in the NC group (Figure 7F).

### Recombinant EIF5A Rescue of the SiRNA-EIF5A Inhibitory Effect

**Figure 8:**
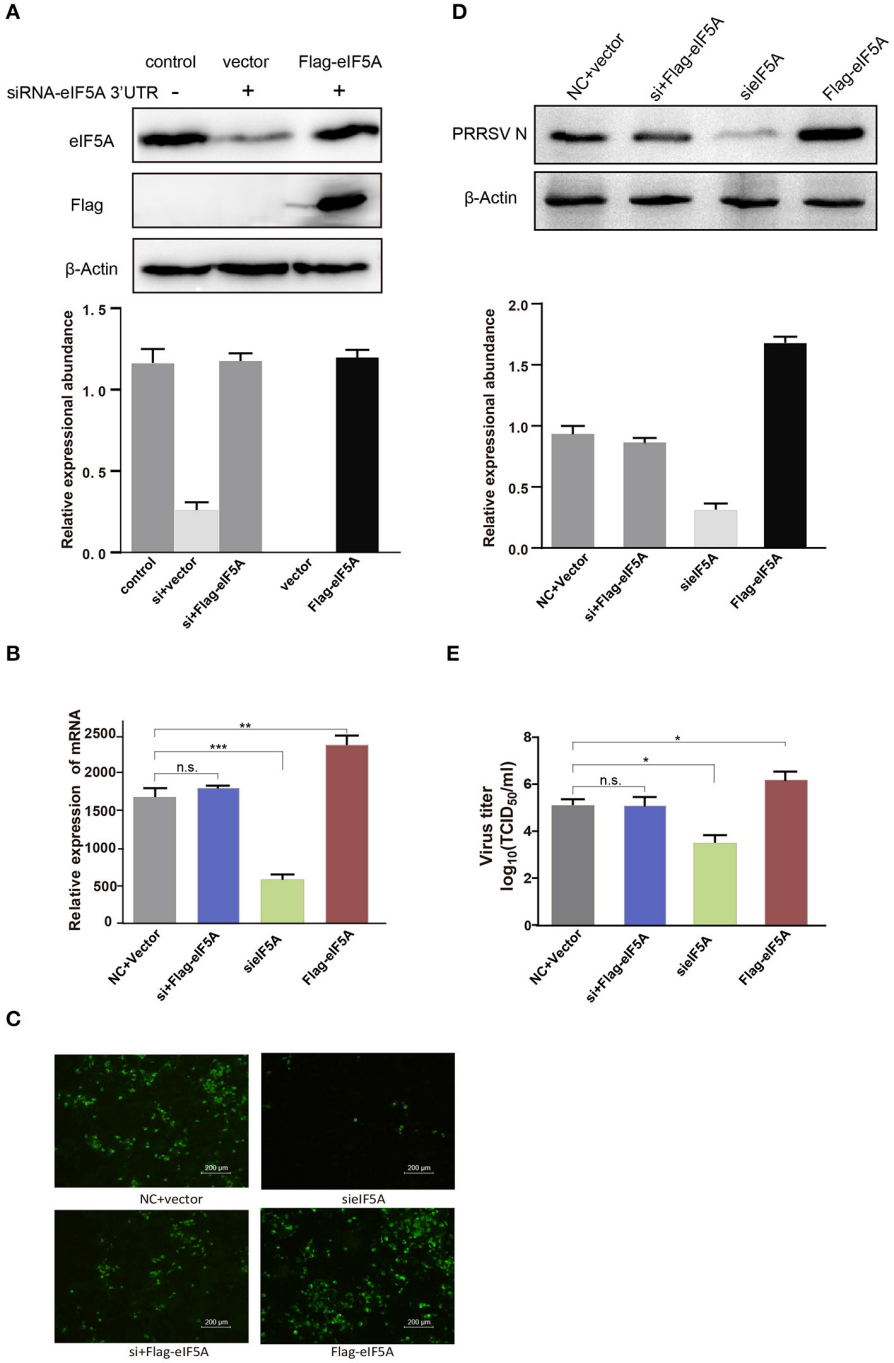
Flag-tagged eIF5A rescued the inhibitory effect of eIF5A knockdown on PRRSV propagation. **(A)** WB analysis of endogenous eIF5A in CRL-2843-CD163 cells and recombinant Flag-tagged eIF5A in CRL-2843-CD163 cells with *eIF5A* knockdown. Endogenous eIF5A was knocked down by siRNA targeting of the *eIF5A* 3′UTR in CRL-2843-CD163 cells. HP-PRRSV HN07-1 (MOI = 0.1) was added to endogenous *eIF5A* knockdown CRL-2843-CD163 cells with recombinant Flag-tagged eIF5A overexpression. PRRSV propagation was validated by **(B)** RT-PCR, **(C)** IFA and **(D)** WB, **(E)** Virus titers were also determined. Each experiment was performed three times independently and all had similar results. *P < 0.05; **P < 0.01; ***P < 0.001; ns, not significant.

To further determine whether overexpression of recombinant *eIF5A* could reverse the *eIF5A* knockdown-induced inhibitory effect on PRRSV infection, we transfected specific siRNA targeting the *eIF5A* 3′UTR into CRL-2843-CD163 cells to knock down endogenous eIF5A expression. Next, the cells were transfected with 3^*^Flag-CMV-eIF5A (Figure 8A) to restore eIF5A expression (as Flag-tagged eIF5A) while inhibiting endogenous eIF5A expression. Thereafter, the modified CRL-2843-CD163 cells were inoculated with HP-PRRSV HN07-1 (MOI = 0.1) then PRRSV propagation was assessed via RT-PCR, IFA, and WB and virus titer determinations. The results revealed that restoration of eIF5A expression in CRL-2843-CD163 cells after *eIF5A* knockdown rescued PRRSV propagation (Figure 8). Meanwhile, cytotoxicity assays showed that cell viability was not adversely affected by siRNA transfection or restoration of *eIF5A* expression (Supplementary Figures 1, 2). Taken together, all of these results demonstrated that eIF5A was required for PRRSV propagation *in vitro*.

## Discussion

PAMs are known target cells of PRRSV infection. Therefore, it is of great significance to study the interaction between PRRSV and PAMs in order to clarify mechanisms involved in viral infection and propagation. Toward this goal, label-free LC-MS/MS can serve as a powerful, quantitative proteomic method that offers many advantages over traditional proteomic methods, including high sensitivity, high coverage, and high accuracy (27, 28). In our study, the proteome of HP-PRRSV HN07-1-infected PAMs was investigated using label-free LC-MS/MS, with uninfected PAMs serving as the control. Ultimately, a total of 269 differentially expressed proteins were identified, among which 46 proteins were significantly up-regulated and 223 proteins were significantly down-regulated (Tables 1, 2).

Importantly, our results revealed that up-regulated proteins were mainly enriched in IFN-I signaling pathways. RT-PCR further confirmed that specific genes associated with these pathways were significantly up-regulated at 24 hpi, such as RIG-I, IFIT1, IFIT3, Mx1, Mx2, STAT1, ISG15, and IFITM1 (Figure 5A). Moreover, expression of several these proteins (IFIT3, Mx1, and STAT1) was confirmed in HP-PRRSV HN07-1-infected PAMs at 24 hpi by WB analysis (Figure 5B). Taken together, these results indicated that PRRSV infection activated the host innate immune system, a result that was consistent with previously reported results. For example, the expression of cytoplasmic virus sensing receptors RIG-I and melanoma differentiation-associated gene five were found to be significantly increased in PRRSV-infected lungs (29). Furthermore, PRRSV infection of MARC-145 cells had been shown to up-regulate expression of Mx2, which when overexpressed was shown to suppress PRRSV replication. In addition, antiviral activity mediated by IFN-β was found to be reduced when Mx2 expression was knocked down, with Mx2 protein observed to reduce PRRSV replication through its interaction with the viral N protein (30). Importantly, infection of PAMs by PRRSV vaccine strains promoted the secretion of extracellular ISG15 from infected PAMs. This observation prompted researchers to introduce recombinant DNA encoding ISG15 into PAMs, after which PAMs expressed ISG15 then entered an antiviral state whereby PRRSV propagation was blocked (31). IFITM3 overexpression had been shown to inhibit PRRSV replication. Meanwhile, endogenous IFITM3 silencing had been shown to promote PRRSV replication. Additionally, it had also been reported that IFITM3 was S-palmitoylated and ubiquitinated and that both of these posttranslational changes contribute to the anti-PRRSV effect of IFITM3 (32).

In contrast to the results mentioned above for up-regulated proteins, down-regulated proteins were mainly enriched for functional terms related to translation-associated and translational initiation-associated processes. More specifically, levels of eIF5A, eIF5B, eIF1A, eIF4H proteins, and ribosomal proteins 40S and 60S were significantly decreased in PAMs after HP-PRRSV HN07-1-infection (Table 2). These results may be explained the host antiviral defense strategy involved the shutting down of translation-related protein synthesis to restrain virus propagation. Indeed, this concept is supported by results of numerous research studies that have shown that levels of eIFs and other host translation-related proteins were significantly decreased in cells after viral infections. For example, after infection with swine transmissible gastroenteritis virus, results of quantitative proteomic experiments revealed that expression levels of eIF3 protein and ribosomal subunit proteins 40S and 60S were considerably decreased in PK-15 cells (33). As another example, during infection with the extremely pathogenic porcine epidemic diarrhea virus (PEDV), the level of eIF2 protein was drastically decreased in Vero cells (34). As yet another example, the host translation system was shown to be repressed after PRRSV infection, with nsp2 and its transmembrane domain found to be responsible for inducing translation shutdown (35). Furthermore, an investigation of proteomic changes associated with organ infection with the PEDV YN144 strain indicated that expression levels of hnRNPA1 and eIF4G1 proteins were decreased in the PEDV YN144-infected group, suggesting that both proteins might be connected to PEDV YN144 strain pathogenicity (36).

Notably, here protein-level expression of eIF5A in PAMs infected with HP-PRRSV HN07-1 was found to be decreased via WB analysis (Figure 6), indicating that this protein might play a role in PRRSV propagation. Meanwhile, after HP-PRRSV HN07-1 infection, eIF5A protein-level expression was down-regulated in PAMs but not in PAMs exposed to UV-inactivated virions, prompting us to speculate that down-regulation of eIF5A expression in PAMs after PRRSV infection was caused by viral replication rather than viral invasion. Interestingly, transcription-level expression of *eIF5A* was not altered in HP-PRRSV HN07-1-infected PAMs at 0, 6, 12, and 24 hpi even though the eIF5A protein level was decreased in HP-PRRSV HN07-1-infected PAMs, warranting further study.

EIFs are important eukaryotic protein translation proteins. EIF2 inhibits protein translation, reduces the levels of early stress proteins and misfolded proteins that are produced in the endoplasmic reticulum (ER), and relieves ER stress (37). Meanwhile, eIF3 mediates ribosome binding to specific RNAs during formation of the translation initiation complex (38), while eIF4E binds to the 5′ methylated cap structure of eukaryotic mRNA and participates in the formation of the translation initiation complex (39). In fact, in recent years results of several studies have shown that eIFs are closely associated with viral replication, including results reported by Regina Cencic *et al*. showing that blocking the functional link between eIF4E and eIF4G greatly decreased replication of human coronavirus (40). In other studies, viral suppression of replication was observed after silencing of eIF4G1 protein expression during infections with vesicular stomatitis virus and influenza virus (41, 42).

EIF5A, which is also known as eIF4D, was first isolated from immature red blood cells (43). It is an acidic protein with a molecular mass of 17–21 kDa that is fairly well-conserved from yeast to humans (44). The function of eIF5A during translation has been widely studied in recent years, with results of studies showing that eIF5A binds to a region of the ribosome that is associated with its translation function, where it acts to promotes the elongation of numerous non polyproline-specific tripeptide sequences (45, 46). EIF5A also binds to 3′-terminal polyadenylation tails of eukaryotic mRNAs and plays a vital role in termination of translation (47). Another critical function of eIF5A is to mediate nucleocytoplasmic transport of mRNA and ensure the balanced distribution of mRNA in the cell nucleus and cytoplasm (48).

In a previous study, eIF5A was observed to play a pivotal role in human immunodeficiency virus (HIV) replication (49). In addition, Ruhl *et al*. reported that eIF5A participated in HIV replication in combination with HIV assistant factor regulator of expression of Rev protein (50). Subsequently, Hofmann *et al*. discovered that Rev protein shuttles back and forth between the nucleus and cytoplasm of host cells as part of its primary function, whereby it controls nuclear transport of non-spliced and incompletely spliced viral mRNAs as an eIF5A-dependent process (51). Meanwhile, mutation of eIF5A has been shown to significantly suppress mRNA nuclear export and inhibit HIV replication *in vitro* without affecting cell propagation and metabolic activity (52). In addition, expression of eIF5A was found to be down-regulated after Fe overload, while expression of the NEF protein of HIV was considerably down-regulated and HIV replication was reduced *in vitro* when eIF5A expression was reduced due to shRNA effects (53). Taken together, these results show that eIF5A plays a role in HIV replication, while eIF5A effects on PRRSV replication have not yet been reported.

Replication of the PRRSV RNA genome is a multi-step process involving the assembly of replication and transcription complexes that consist of viral and cell-derived components (54–56). In this study, we first investigated the role of eIF5A in PRRSV replication *in vitro*. Notably, PRRSV propagation was significantly inhibited after knockdown of *eIF5A* expression in CRL-2843-CD163 cells and PAMs even though *eIF4E* knockdown did not affect viral propagation (Figure 7). However, suppression of HP-PRRSV infection after *eIF5A* knockdown could be reversed by restoration of host cell expression of eIF5A (Figure 8). Although these results are intriguing, they raise additional questions regarding the mechanisms underlying eIF5A involvement in PRRSV propagation. Thus, experiments are currently underway in our laboratory to answer these questions toward the development of novel anti-viral strategies and more effective anti-viral drugs to combat PRRSV.

## Conclusion

In summary, here dynamic changes in the proteome of HP-PRRSV HN07-1-infected PAMs were analyzed using label-free LC-MS/MS techniques, resulting in identification of a total of 269 significantly differentially expressed host proteins. Interestingly, expression of one of these proteins, eIF5A, was down-regulated in PAMs after HP-PRRSV HN07-1 infection, while PRRSV replication was significantly inhibited and the viral titer was suppressed considerably by *eIF5A* knockdown *in vitro*. Taken together, these results demonstrated that eIF5A participates in PRRSV infection and created a foundation for further exploration of mechanisms toward the development of antiviral strategies to control and prevent PRRSV infection.

## Data Availability Statement

The datasets presented in this study can be found in online repositories. The names of the repository/repositories and accession number(s) can be found in the article/Supplementary Material.

## Ethics Statement

The animal study was reviewed and approved by The Ethical and Animal Welfare Committee of the Key Laboratory of Animal Immunology of the Ministry of Agriculture of China.

## Author Contributions

HL, RL, and SQ designed the experiments. HL, BW, DJ, PJ, MZ, and XL performed the experiments and analyzed the data. HL wrote the paper. RL and SQ revised the article, and all authors approved the final manuscript.

## Funding

This study was supported by grants from National Natural Science Foundation of China (31902284, 31902279), Funding scheme for young teachers in colleges and universities in Henan province (2020GGJS258), Doctoral Research Initiation Fund of Henan University of Animal Husbandry and Economy (2019HNUAHEDF040).

## Conflict of Interest

The authors declare that the research was conducted in the absence of any commercial or financial relationships that could be construed as a potential conflict of interest.

## Publisher's Note

All claims expressed in this article are solely those of the authors and do not necessarily represent those of their affiliated organizations, or those of the publisher, the editors and the reviewers. Any product that may be evaluated in this article, or claim that may be made by its manufacturer, is not guaranteed or endorsed by the publisher.
