# Supplementary material for: Proteomic Investigation Reveals Eukaryotic Translation Initiation Factor 5A Involvement in Porcine Reproductive and Respiratory Syndrome Virus Infection in vitro
Source: Front Vet Sci. 2022 Apr 13;9:861137. doi: 10.3389/fvets.2022.861137 (PMC9043857; doi:10.3389/fvets.2022.861137)

**Supplementary Figure 1: SiRNA transfection does not affect cell viability.** CRL-2843-CD163 cells were transfected with the indicated siRNAs targeting eIF5A, and eIF4E at a final concentration of 0.2 mM using Lipofectamine RNAiMAX. Non-targeting siRNA was used as negative control. The cytotoxicity experiment was conducted at 24, 36 and 48 h after siRNA transfection by adding MTS reagent to the cells, followed by incubation at 37 °C for 1 h. Absorbance was measured at 490 nm.


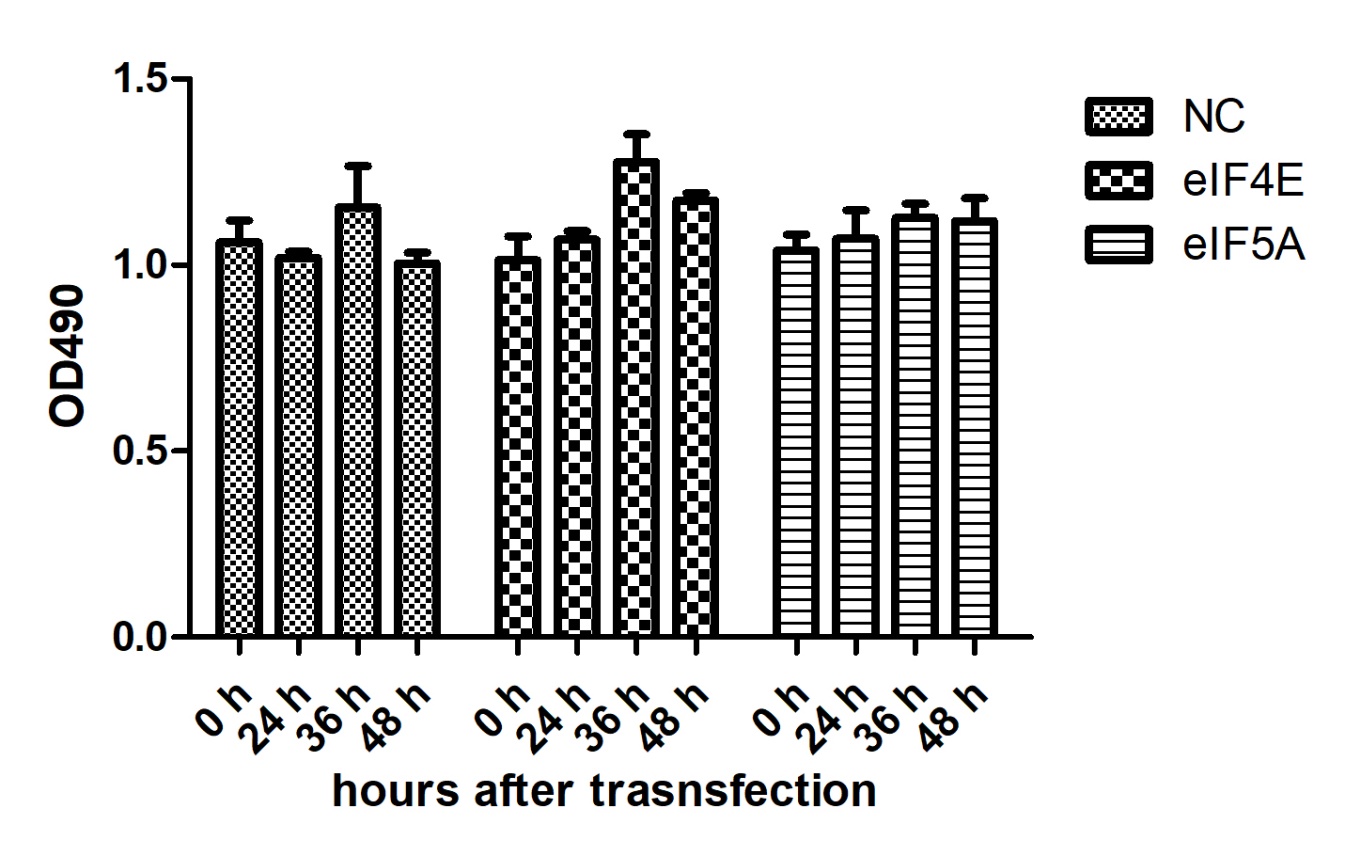


**Supplementary Figure 2:** The eIF5A rescue experiment does not affect cell viability. CRL-2843-CD163 cells were transfected with the siRNAs targeting eIF5A 3’UTR. The cytotoxicity was also conducted 48 h after 3*Flag-CMV-eIF5A transfected into *eIF5A*-knock down CRL-2843-CD163 cells using Lipofectamine LTX plus. The cytotoxicity experiment was conducted as described above.


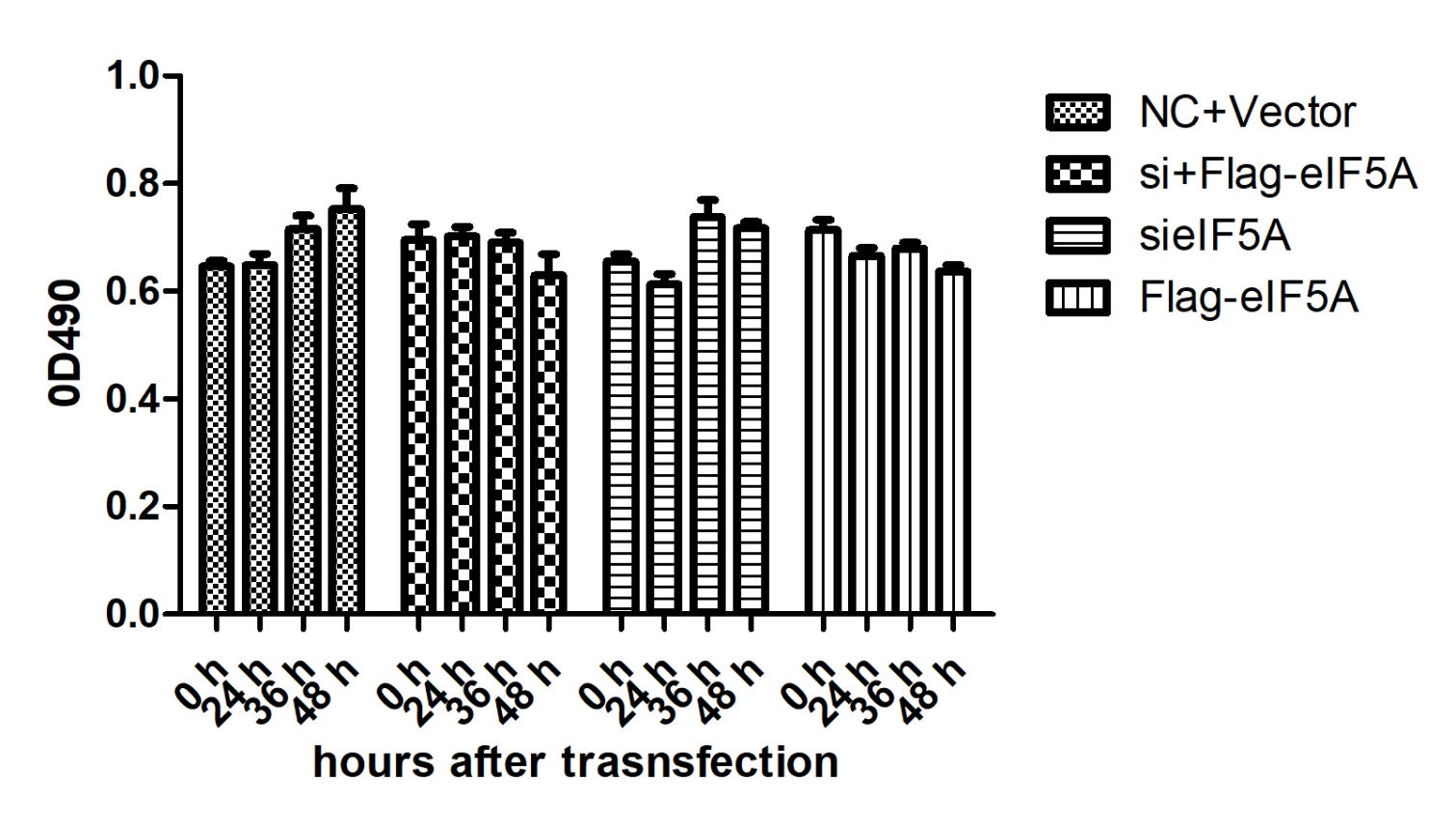

Supplement: Supplementary file 2 [file Data_Sheet_1.DOCX]
